# Evaluating the effects of episodic and semantic memory induction procedures on divergent thinking in younger and older adults

**DOI:** 10.1371/journal.pone.0286305

**Published:** 2023-06-02

**Authors:** Halima Ahmed, Kata Pauly-Takacs, Anna Abraham

**Affiliations:** 1 School of Humanities and Social Sciences, Leeds Beckett University, Leeds, United Kingdom; 2 Department of Educational Psychology, Mary Frances Early College of Education, University of Georgia, Athens, GA, United States of America; 3 Torrance Center for Creativity and Talent Development, Mary Frances Early College of Education, University of Georgia, Athens, GA, United States of America; University of Trieste, ITALY

## Abstract

Evidence suggesting that episodic specificity induction improves divergent thinking performance in younger and older adults has been taken as indicative of the role of declarative memory processes in creativity. A series of studies were carried out to verify the specificity of such findings by investigating the effects of several novel episodic and semantic memory induction procedures on a widely employed measure of divergent creative thinking (the Alternate Uses Task), in comparison to a control induction and a no-induction baseline in both younger and older adults. There was no clear evidence for a specific role played by the induction of episodic or semantic memory processes in facilitating creative thinking across the three experiments, and the effects of the induction procedures (episodic, semantic and control) on divergent thinking were not comparable across age groups. On the other hand, higher levels of creativity were generally associated with older adults (60–80 years). In Experiments 2 and 3, older adults generated a greater number of responses (fluency), more unique responses (average originality, peak originality, creativity ratings) and more varied responses (flexibility) than younger adults (18–30 years). The findings are discussed in relation to the specificity of declarative memory operations and their impact on creative thinking, especially within the context of healthy ageing.

## Introduction

The relationship between creativity, memory and ageing is a perplexing one. While healthy ageing is associated with a decline in several cognitive abilities, creative capacity seems to be relatively preserved. Creativity has been the subject of extensive research over several decades. However, the cognitive underpinnings of creative thinking are, as yet, not well understood. The relationship between creative thinking and declarative memory operations, such as episodic memory retrieval ability has received much focus of late [1–6]. Madore, Addis and Schacter [7] postulated that if a cognitive task relies on episodic memory, performance on the task should improve following the induction of episodic-specific processes. They implemented a novel approach, an *episodic specificity induction* (ESI), which consisted of a brief training in the viewing of an event and then recollecting specific details of that event directly after. The ESI is held to boost new idea production by targeting retrieval of prior episodic experiences that are recombined to then create novel episodic representations [8]. They argued that the ESI biases the *retrieval orientation* adopted in tasks, such that people focus more on episodic details pertaining to people, objects, and actions.

This is in line with the *constructive episodic simulation hypothesis* [9], which has been described as the ability to flexibly recombine elements of past experiences to form novel episodic representations. A widely used divergent thinking measure, the Alternate Uses Task [AUT; 10] was employed to test creativity, in which participants were asked to list as many unusual or creative uses as possible for common objects. Compared to non-episodic control procedures, they found that the ESI significantly enhanced some facets of divergent thinking (fluency and flexibility), but had no impact on others (originality, elaboration). A similar boosting effect of the ESI was also observed in relation to another divergent thinking task, the Consequences Task, in which participants were asked to list consequences to a hypothetical event (e.g., what if people no longer needed to sleep) [8].

They then explored the same idea in relation to age-related differences [8]. Both younger and older adults showed significantly greater fluency and flexibility (but not originality) following the ESI compared to a control induction. This is noteworthy because there is evidence to suggest that creative skills appear to be relatively preserved in healthy older adults [11–13]. In a recent systematic review, Fusi et al. [14] considered 16 studies that examined the effect of age on divergent thinking. Whilst there were substantial inconsistencies in the findings across studies owing to procedural differences and measures used, the review highlighted that verbal divergent thinking abilities in older adults were preserved if limits on processing speed and working memory capacity were taken into account within the study design. On the other hand, a decline in episodic memory function (but unimpaired semantic memory) is typically associated with healthy ageing [15–17]. One of the challenges is therefore to understand how best to integrate these findings given that older adults benefit from ESI on various cognitive tasks, such as means-end problem-solving [18].

Gaining an insight into what specific aspects of episodic retrieval processes aid creative ideation is central to understanding the benefits of such memory inductions. The very definition of ‘episodicity’, or what makes an induction ‘episodic’ in nature, is critical in understanding which components of episodic memory may be facilitating creative ideation. While there is general consensus about what episodic memory entails, there are slightly different working definitions of it, producing differences in the criteria used to study it, and inconsistencies in how episodic memory is tested [19]. The standard definition of episodic memory concerns the ability to recollect specific *personal* experiences that happened in a particular time and place through ‘mental time travel’ [20, 21], whereas other conceptualisations concern the recall of the ‘what’, ‘when’ and ‘where’ elements of an event that is not necessarily personal [22]. The early work of Tulving [23, 24] made no conceptual distinction between the concept of episodic memory and autobiographical memory (i.e., memory for personal life events) [25, 26], and the interchangeable use of these two concepts has been debated in the memory literature ever since [27].

The difference between autobiographical memory and lab-based tasks of episodic memory in terms of processing demands is one to be noted. For example, while autobiographical retrieval can span decades, stimuli in laboratory-based tasks of episodic memory (e.g., word occurrence in a list) are typically presented only a short time before the test [28]. Furthermore, an investigation of several laboratory-based tests of episodic memory by Cheke and Clayton [29] has indicated that not all these tasks result in comparable performance. Three episodic memory tasks were considered; the What-Where-When (WWW) task in which participants were asked to hide coins of different types at different times and later asked to indicate the location of a particular coin which they hid at a particular time; the Source Memory (SM) task in which participants were asked a series of questions relating to contextual/incidental features of the experiments; and the Free/Cued Recall (FR) task in which participants were presented with a word list that they had to recall after a delay. Correlational analysis revealed inconsistencies in performance. For example, participants who performed well on the WWW task performed poorly on the SM task, suggesting that not all these tests of episodic memory are necessarily tapping the same processes [30].

Similarly, differences have been identified in the neural networks supporting laboratory-based tasks of episodic memory and autobiographical memory tasks, such that the parietal memory network and the fronto-parietal control network were preferentially engaged during the former, while the default mode network was preferentially engaged during the latter [30]. Scene construction or “the process of mentally generating and maintaining a complex and coherent scene or event” [31, p. 299] has also been closely linked to episodic memory such that it engages similar hippocampal regions associated with remembering the past and imagining future events [31, 32]. A neuropsychological study [33] involving participants with medial-temporal lobe pathology and resulting episodic-autobiographical memory loss has shown negative consequences of the impairment for solving open-ended problems, which are similar to divergent thinking tasks in that they do not require a single correct answer. This issue is of relevance here because the study of the influence of episodic memory processes on divergent thinking has seen the use of different procedures, some involving the induction of impersonal episodic memories [e.g., 7] while others have relied on the induction of autobiographical memories [e.g., 34].

Therefore, to gain an insight into the specific aspects of ‘episodicity’, which when induced is expected to result in a biasing effect on the retrieval orientation adopted in subsequent tasks, it is necessary to explore alternative episodic inductions that tap various aspects of ‘episodicity’.

Several interesting questions arise in this context. Would positive induction effects on creativity result with an alternative procedure that taps other elements of episodic retrieval processes? Another related question is whether the effects of a memory induction are specific to episodic memory, or whether they can be extended to other forms of declarative memory, such as semantic memory, which has traditionally occupied a central focus in relation to creative thinking [34, 35]. For example, studies that have examined which problem-solving strategies are adopted while generating alternative uses for familiar objects in divergent thinking tasks have reported that the use of experiential strategies (i.e., where specific experiences are invoked, and thus likely draw on episodic memory) predominate in the early processes of generation. By contrast, abstract semantic-based strategies (e.g., imagined disassembly of the target object) are dominant in later processes of generation. Importantly, originality and fluency were predicted by memory strategy, such that the use of more effortful and executively demanding semantic-based strategies led to more novel responses [36]. In line with these findings, the behavioral results from one recent study examining the effects of different memory inductions (i.e., semantic and episodic) on divergent thinking found that semantic distance was significantly larger following semantic induction compared to episodic induction, indicating that the semantic induction increased the creativity/originality of responses [34].

Individual differences in creative thinking have also mainly centred on semantic cognition-relevant operations to date [37, 38]. For instance, Mednick’s [39] associative theory differentiates low and high creative individuals by characterising them as having distinctive knowledge associative hierarchies. The activation of remote associations is held to allow for the generation of alternative solutions and is therefore vital in divergent thinking [40]. More ‘original’ or high creative individuals have broader semantic networks than low creative individuals [41–43]. Moreover, semantic distance between responses and the concept presented on a divergent thinking task significantly predicts subjective creativity ratings in a positive direction [44]. Lesion-based approaches in examining divergent thinking in semantic dementia patients (SD) and behavioral-variant fronto-temporal dementia patients (bvFTD) compared to neurotypical control participants revealed that while both SD and bvFTD patients provide fewer responses and less original responses compared to the control group, SD patients perform worse on the fluency measure compared to bvFTD patients [45]. Taken together, these findings demonstrate differences in creative ability as a result of differences in semantic cognition-relevant operations, highlighting a critical role of such operations for creative cognition.

Prior research has largely demonstrated well-preserved semantic memory and stability of semantic knowledge in healthy cognitive ageing [46, 47]. Using percolation analysis to examine the robustness of semantic networks, Cosgrove et al. [48] showed that whilst older adults retain stability in the size of their semantic network structure, their semantic networks broke down faster and are less flexible in comparison to younger adults. Using the same approach to analyse the semantic networks of high and low-creative groups, Kenett et al. [42] showed that the semantic network of the high-creative group was more connected and less segregated compared to the low-creative group, indicating heightened flexible thinking. It is therefore important to also explore how semantic memory processes may impact creativity as a function of ageing. In light of evidence suggesting the benefit of the ESI on divergent thinking in older adults [8] and the contribution of semantic memory in creative idea generation [34] it is possible that such effects of a semantic induction could also be extended to older adults.

The present paper reports a series of experiments designed to investigate whether performance is enhanced on the AUT in younger and older adults following an episodic and/or semantic induction, compared to a control induction and a ‘no-induction’ baseline condition. Each experiment saw the focus on different aspects of episodicity. The no-induction condition, in which no task was carried out prior to completing the AUT, was implemented in order to determine the direction of change associated with the episodic and semantic inductions (better, worse, or undifferentiated from baseline). To date, no prior studies have incorporated a true no-induction baseline. Madore et al. [7, 8] implemented a control math induction which they referred to as a ‘neutral baseline’. Episodic and semantic memory inductions were implemented within the same paradigm in order to evaluate whether the effects of memory retrieval operations on the key components of creative ideation (fluency and originality) are limited to declarative operations that are either episodic or semantic in nature. In order to make the declarative memory inductions as comparable in nature as possible within each experiment, it was necessary to devise a series of novel inductions different from that of the original by Madore and colleagues [3]. Experiment 1 and 2 therefore implement novel forms of episodic and semantic memory inductions, while Experiment 3 adopts the ESI procedure introduced by Madore et al. [3].

In summary, the present series of experiments aims to explore the idea that if creativity is enhanced by episodic and/or semantic memory retrieval operations, inducing such declarative memory operations should enhance creative performance for both younger and older adults, compared to a control non-declarative memory induction and no-induction baseline. A number of predictions were tested in light of the consistent pattern of findings that have been evidenced in the literature. These are discussed in the subsequent introductory sections for each experiment.

An important question for exploration is whether there is a differentiated effect of the two declarative memory induction types on creativity. It is possible that each induction type influences different aspects of divergent thinking (e.g., episodic induction influences fluency versus semantic induction influences originality), as suggested by Madore et al. [7] and Beaty et al. [34] studies, as well as divergent problem-solving strategy-based evidence [36].

## Experiment 1

### Introduction

The episodic induction in Experiment 1 targeted the induction of episodic memory by engaging autobiographical as well as event construction processes. As personal event-based memory retrieval was selected as the key component of episodicity to focus on within Experiment 1, the episodic induction in this experiment entailed retrieval of detailed personal life *events*. A well-established autobiographical memory task was employed to evoke detailed recall of personal past events using a cue word [49].

To maintain comparability between the episodic and semantic inductions with regard to the task characteristics (i.e., presentation of cue words) the semantic induction consisted of a free association task, commonly used in semantic memory research [41, 50]. A similar free word association task on which highly creative individuals have demonstrated significantly higher associational fluency [41], has been adopted in a previous study to understand the link between semantic processes and creativity. Beaty et al. [34] implemented a semantic induction task to assess the impact of semantic operations on divergent thinking by presenting participants a cue word to which they were required to first generate a word that was closely associated to it, followed by a sentence including those two words. This resembles the associative component of the semantic induction task adopted in the current experiment. Finally, a within-subjects no-induction baseline trial was adopted in order to be able to effectively compare differences in divergent thinking following inductions. We predict that creative performance will be enhanced for both younger and older adults following the episodic and semantic inductions compared to the control induction. It is also hypothesised that the episodic and semantic inductions will result in greater creative performance compared to the no-induction baseline for both younger and older adults.

### Materials and methods

#### Participants

Thirty younger adults (18–30 years, M = 21.93, SD = 3.69; 23 women, 7 men) and thirty older adults (60–80 years, M = 70.63, SD = 4.48; 22 women, 8 men) participated in the experiment. Participants were recruited from the first author’s institution and the local area. They received either course credits or a shopping voucher for their participation. All participants were fluent English speakers and had no history or diagnosis of a neurological, neurodevelopmental, or psychiatric condition. All participants provided written informed consent prior to participation. The study was approved by the Local Psychology Research Ethics Committee of the first author’s institution. Older adults completed the Mini Mental State Examination (MMSE) in the screening process and only those with a score of 25 or above were included in the sample [51]. The 9-item short Form A of the Raven’s Standard Progressive Matrices (RSPM) [52] was also administered to all participants to ensure they fell within the normal range of IQ scores. As there was a marginally significant difference between the IQ scores of younger (*M* = 104.87, *SD* = 8.79) and older adults (*M* = 100.27, *SD* = 9.42) (*t*(58) = 1.96, *p* = .055, *d* = .52), IQ was entered as a covariate in the statistical analyses (i.e. ANCOVAs were performed).

#### Tasks

*1*. *Episodic induction*. The Galton-Crovitz cueing technique [53] was used to elicit episodic retrieval in the episodic induction. Ten cue words were selected from Janssen, Rubin and Jacques [54] (e.g., mountain, storm, coffee). The length of the induction was 5 minutes. Participants varied in the number of cue words used to take up 5 minutes of retrieval phase (range: 1–5 words). Participants were presented words in a random order, one after the other. The instructions provided were as follows:

“This task is about your personal memories. You will be presented with a set of words, one by one, and asked to describe the first memory that comes to mind associated to that word. The memory can be from any part of your life and does not have to be interesting but must be specific and last no longer than one day. For example, if I present you with the word *blossom*, ‘spring’ would be an incorrect response as would be ‘a holiday that you went on last spring’, but a description of a specific event that occurred on a spring day or on this holiday would be correct. On the sheet in front of you, you can see a series of questions. I would like you to think about these when recalling the memory. For example, *What happened*? *What did you do*? *How did you feel*? *Whom was there*? You will also be asked to give an approximate date for when this event occurred, so please try to describe the memory in as much detail as possible. Any questions?” Along with the above questions, the list of prompt questions also included the following: *Where it happened*? *How it happened*? *What was the weather like*? *What was the atmosphere like*?”

Participants were asked to indicate if they could not recall a specific memory associated with the cue word presented, and if so, they were presented with another word. The questions above were also provided in written form to aid retrieval and an example memory description was provided. A maximum of two prompts were used to further aid detailed recall. Once 5 minutes elapsed participants were asked to stop.

#### Episodic induction scoring

Responses were audio recorded and transcribed. For the episodic induction ‘responsivity scoring’ (i.e., a measure of engagement with the induction task), the Text Segmentation and Categorisation scoring technique from Levine et al. [16] was adopted. Responses were scored for internal and external details. Episodic details pertaining to a specific event, time, place, and those reflecting episodic re-experiencing, including perceptual details and thoughts and emotions, were classified as ‘internal details’. ‘External details’ consisted of semantic information, repetition and references to external events. Each response was segmented into informational details expressed as grammatical clauses and all pieces of information were given an internal or external score, resulting in an internal to external ratio. For example, a response with 57 details in total, consisting of 32 internal details and 25 external details resulted in a score of .56, indicating 56% of details being internal. Two raters scored 12 responses (20% of all responses) and obtained good interrater reliability (Cronbach’s α = .79). Raters were blind to which age-group the memory descriptions came from.

*2*. *Semantic induction*. A word association task was employed for the semantic induction. Ten cue words were taken from Gianotti [55]. The length of the induction was 5 minutes. Participants varied in the number of cue words used to take up 5 minutes of induction length (range: 4–12 words). Cue words were presented in a random order. When the time responding to one word had elapsed, the next word was presented. The instruction provided were as follows [modified from 37]:

“This is a word association task. In front of you, you will see a single word. Please think of as many related responses to this word you can think of. I will let you know when to move on to the next word. We will begin with me providing you an example and then move on to the task. For example, for the cue word ‘flower’ you may think of the following responses, *garden*, *sun*, *spring*, *water*, *roses*, *butterflies*, *park*, *etc*. Please provide your responses out loud.”

#### Semantic induction scoring

The number of associations for each presented word were calculated and computed into an average semantic association ‘responsivity score’ across the number of words presented. For example, if a participant provided a total number of 32 responses across 4 cue words, they would obtain an association responsivity score of 8. There was a significant difference in the number of cue words employed in the semantic induction by older adults (*M* = 5.2, *SD* = .89) and younger adults (*M* = 7.4, *SD* = 1.94) (*t*(58) = 5.65, *p* < .001, *d* = 4.72), along with a significant difference in the number of cue words employed in the episodic induction by older adults (*M* = 2.37, *SD* = .81) and younger adults (*M* = 3.33, *SD* = 1.45) (*t*(58) = 3.19, *p* < .01, *d* = 1.68). This was not considered an issue as the length of induction remained constant at 5 minutes for all participants. Given that it was necessary to control for the length of time participants engaged in the inductions, it was expected that there may be differences in the number of cue words required to reach the 5-minute time limit.

*3*. *Control induction*. A non-verbal and non-declarative memory task was used as a control induction. Participants were presented with a simple math operation (e.g., +7–3) and the first three digits from a number sequence (e.g., 2, 6, 10). The control induction consisted of five 60-second trials. For each trial participants were instructed to continue calculating the next digit in the sequence using the operation provided until the 60 seconds had elapsed. The purpose of a non-verbal task in the control induction was to avoid any word cues evoking episodic or semantic retrieval operations.

#### Control induction scoring

The average number of responses given across the three trials constituted the control induction ‘responsivity score’.

*4*. *Alternate uses task*. The AUT [10] was used as a test of divergent thinking. Participants were required to think of as many alternate uses as they could for a common object. For example, the common use for a ‘pencil’ is for writing, but it can be used as a toy drumstick. The AUT consisted of four trials: one no-induction baseline trial and one trial following each of the three induction conditions. The objects included were *fork*, *car tyre*, *hanger* and *belt*, and were presented to participants in written form. Previous research has proposed that older adults often take longer completing a divergent thinking task [11, 56]. Therefore, to maintain the timing to be sufficient and standardised across groups, a period of five minutes was given to think of as many alternate uses as possible in each trial. Participants were instructed to think of as many creative uses as they could. Responses were expressed verbally by the participant while the researcher wrote them down. This allowed the researcher to ask participants to explain the idea of the use, if necessary. One prompt was given to all participants upon indication of exhausting response output (“Can you think of any more uses? You still have time”). Of the four measures of divergent thinking that were evaluated, three assess originality in divergent thinking. The reason for adopting this approach was primarily because of the great variability in how originality is assessed across studies. Using three different protocols and examining the commonalities and differences in the findings associated with them would allow for clearer generalisations to be made about differences in originality (the defining attribute of creativity) as a function of the inductions and/or age.

#### AUT scoring

Prior to scoring the AUT all responses were screened to determine whether they were appropriate/inappropriate. As stated in the AUT manual, “A use, to be acceptable, should be possible for the object” [10, p. 30]. Hence, only the responses deemed appropriate were further scored on the measures of the AUT [10].

(a) *Fluency scoring*: This refers to the total number of appropriate uses that were generated for each object.
*(b) Average originality scoring (objective)*: Originality scores reflect the uniqueness associated with the generated uses. In this study, originality was calculated using three methods. The first method considered an overall originality score including all the items generated. This average originality score was calculated from the proportional weighting of each use by the frequency of its occurrence [57, 58]. To maintain high originality as indicative of high divergent thinking, and to resolve the confounding influence of fluency on originality (i.e., those with high fluency score having a high originality score as a result of more uses being summed in the calculation) [59], this single score was then divided by the fluency score of the participant and subtracted from 1 (e.g., 1 - (.42/4)), resulting in a difference score (e.g., .90), which was referred to as the average originality score.
*(c) Peak originality scoring (objective)*: This method only considered responses of high originality as evidenced by the statistical rarity of the responses within the sample [57, 58]. The proportional weighting (i.e., number of times the use was generated in the sample / total number of participants) of each use was converted into a percentage (e.g., 0.17 = 17%). Only uses with a proportional weighting of 10% or less (generated by less than 10% of the sample) were considered. The number of uses generated by each participant that fell within this 10% were summed and resulted in a peak originality score. For example, if a participant had generated 4 uses that were generated by less than 10% of the sample, the person would have a peak originality score of 4.
*(d) Subjective creativity ratings*: The subjective creativity rating method was also adopted as a measure of novelty/originality. Three trained independent raters evaluated the creativity of all the uses generated for all the objects on a scale of 0 (not creative) to 4 (highly creative). They were informed that their creativity judgements should consider both the originality and the appropriateness of the use and that uses can be considered highly creative if they are original, useful, and presumably only a few people would provide that use [35, 60]. The raters showed good interrater reliability (Cronbach’s α = .84). The creativity rating of a single idea was calculated by obtaining the average creativity rating given by the three independent raters. The final creativity rating for each participant was then calculated by generating an average of their creativity ratings after each induction condition (i.e., sum of the ratings for all uses divided by the number of uses generated).

*5*. *RSPM–Short form A*. This 9-item scale is an abbreviated version of the 60-item Raven’s Standard Progressive Matrices (SPM) [52]. The SPM is a non-verbal test measuring intellectual capacity. Participants are required to complete an incomplete pattern provided, by choosing the one correct option that would accurately complete the pattern from 6–8 potential options. IQ scores were calculated using the formula provided in Bilker et al. [52].

#### Experimental design and procedure

Participants attended a single session for testing and took part in all induction conditions; no-induction baseline, episodic induction, semantic induction, and a control induction. A within-subjects design was deemed to be appropriate to closely follow the design of those used in prior studies [e.g., 7, 8], and in light of evidence from ageing-related research indicating greater individual variability in performance on cognitive tasks in older adults [61]. The order in which participants received the inductions, beside the no-induction baseline, was counterbalanced, as was the order of items within the inductions. All participants completed the no-induction baseline AUT prior to receiving any other induction. The AUT trials following each induction were also counterbalanced. Participants were randomly assigned to the specific induction/AUT order they received, with a balanced number of participants in each order-group (n = 18–19 per order group). After completing the no-induction baseline AUT, participants received the first induction, following which they immediately completed one trial of the AUT. Participants then took part in a filler task (Dot to Dot puzzle) for 5 minutes. The above procedure was then repeated for the remaining two induction conditions. At the end of the experiment, all participants completed the RSPM and were then debriefed. The session took approximately 70 minutes to complete.

### Results

#### Induction responsivity

**Fig 1:**
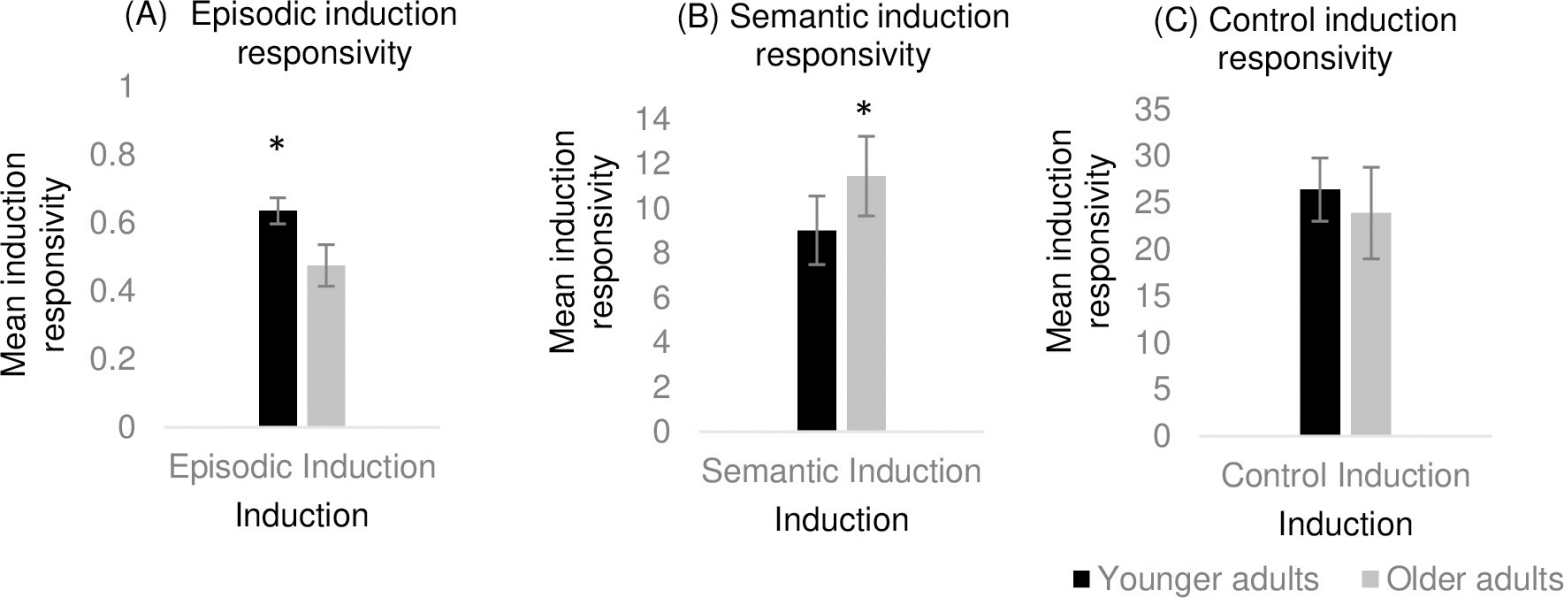
Experiment 1 findings. Mean induction responsivity score in each induction task for young and older adults. Error bars represent 95% CI. *Note*. * Significant difference between younger and older adults (*p* < .05).

A series of one-way ANCOVAs were conducted to establish age-based differences across induction responsivity. There was a significant difference between younger and older adults on the episodic induction responsivity (*F*(1, 58) = 20.83, *p* < .001, η^2^ = .26), such that younger adults (*M* = .64, *SD* = .10) generated significantly more internal details than older adults (*M* = .48, *SD* = .16) (Fig 1A). There was also a significant difference between younger (*M* = 9.03, *SD* = 4.11) and older adults (*M* = 11.45, *SD* = 4.17) in semantic induction responsivity (*F*(1, 58) = 5.16, *p* = .027, η^2^ = .09), such that older adults generated a greater number of associations than younger adults (Fig 1B). There was no significant difference between younger and older adults in the control induction responsivity (*F*(1, 58) = 2.98, *p* = .089, η^2^ = .04) (Fig 1C). In sum, there was a dissociation in the pattern of induction responsivity such that younger adults showed greater responsivity following the episodic induction whereas older adults demonstrated stronger responsivity following the semantic induction.

#### Order-based differences

To check for task/induction order-based differences prior to the main statistical analyses, a series of one-way ANCOVAs were conducted. The findings revealed no significant difference on fluency (*F*(2,57) = 1.56, *p* = .218, η^2^ = .05), average originality (*F (*2,57) = 3.19, *p* = .728, η^2^ = .01), peak originality (*F*(2,57) = 1.18, *p* = .314, η^2^ = .04), and subjective creativity ratings (*F*(2,57) = .833, *p* = .440, η^2^ = .03) as a function of induction order or AUT order: fluency (*F*(3,56) = .831, *p* = .483, η^2^ = .04), average originality (*F*(3,56) = 1.61, *p* = .198, η^2^ = .08), peak originality (*F*(3,56) = .773, *p* = .514, η^2^ = .04), and subjective creativity ratings (*F*(3,56) = .454, *p* = .716, η^2^ = .02). This confirms that there was no effect of the order in which participants received the induction, and no effect of the order in which participants received the AUT objects after each induction on the four metrics of creative thinking.

#### Differences following episodic, semantic, and control inductions

*A* series of 3 (Induction: episodic, semantic, control) × 2 (Age: younger vs older) ANCOVAs were conducted in relation to all four AUT measures (see Table 1 for descriptive data). With regard to fluency, there was no main effect of induction (*F* (2, 114) = .071, *p* = .932, η_p_^2^ = .001), age (*F* (1,57) = .144, *p* = .705, η_p_^2^ = .003), or age × induction interaction (*F* (2, 114) = .575, *p* = .564, η_p_^2^ = .010).

**Table 1:** Means (M) and standard deviations (SD) for the alternate uses task (AUT) in Experiment 1.

	No-induction baseline		Episodic induction		Semantic induction		Control induction	
	Younger adults	Older adults	Younger adults	Older adults	Younger adults	Older adults	Younger adults	Older adults
AUT measures	*M (SD)*	*M (SD)*	*M (SD)*	*M (SD)*	*M (SD)*	*M (SD)*	*M (SD)*	*M (SD)*
Fluency	7.1 (4.96)	7.7 (4.65)	7.43 (5.87)	6.63 (4.19)	7.16 (4.76)	5.83 (3.63)	7.06 (4.21)	6.5 (3.48)
Average originality	.74 (.11)	.78 (.09)	.75 (.11)	.75 (.11)	.74 (.08)	.69 (.08)	.78 (.08)	.72 (.1)
Peak originality	2.3 (3.03)	2.93 (3.04)	2.97 (4.07)	2.6 (2.94)	2.1 (2.86)	1.27 (1.94)	2.43 (2.37)	1.77 (1.96)
Subjective creativity ratings	2.01 (.35)	2.08 (.35)	1.95 (.48)	2.09 (.28)	1.96 (.38)	1.99 (.39)	2.01 (.31)	2.05 (.31)

In sum, there were no differences in the performance of younger and older adults on the AUT, and none of the inductions had a significant impact on any measured aspect of divergent thinking.

With regard to the three originality measures, there were no significant effects in relation to average originality (induction: *F*(2, 114) = 2.59, *p* = .08, η_p_^2^ = .044; age: *F*(1, 57) = .155, *p* = .218, η_p_^2^ = .026; age × induction interaction: *F*(2, 114) = .1.95, *p* = .147, η_p_^2^ = .033), peak originality (induction: *F* (2, 114) = .994, *p* = .373, η_p_^2^ = .017; age: *F*(1,57) = .264, *p* = .609, η_p_^2^ = .005; age × induction: *F*(2, 114) = .656, *p* = .521, η_p_^2^ = .011), or subjective creativity ratings (induction: *F*(2, 114) = 1.06 *p* = .35, η_p_^2^ = .018; age: *F*(1,57) = 2.42, *p* = .126; η_p_^2^ = .041, age × induction: *F*(2, 114) = .794, *p* = .454, η_p_^2^ = .014).

#### Analyses in relation to the no-induction baseline

A series of one-way ANCOVAs were carried out to assess whether younger and older adults’ no-induction baseline scores on the AUT differed on the four creativity measures. There was no significant difference between younger adults and older adults on any of the dependent measures: baseline fluency (*F*(1, 58) *=* .233, *p* = .631, *d* = .12), baseline average originality (*F*(1, 58) *=* 1.53, *p* = .221, *d* = .27), baseline peak originality scores (*F*(1, 58) *=* .653, *p* = .422, *d* = .19), and baseline subjective creativity rating (*F*(1, 58) *=* .703, *p* = .405, *d* = .2). This suggests that both younger adults and older adults perform comparably on the AUT when there is no induction implemented prior to completing the task.

A series of paired samples t-tests were conducted to assess whether performance on the AUT was significantly different following the episodic, semantic, and control inductions in comparison to a no-induction baseline.

Younger adults showed no significant differences for fluency between the no-induction baseline and following episodic induction (*t*(29) = -.483, *p* = .633, *d* = .06), semantic induction (*t*(29) = -.115, *p* = .909, *d* = .01), and control induction (*t*(29) = .066, *p* = .948, *d* = .008) (pbonferroni = .017).

**Fig 2:**
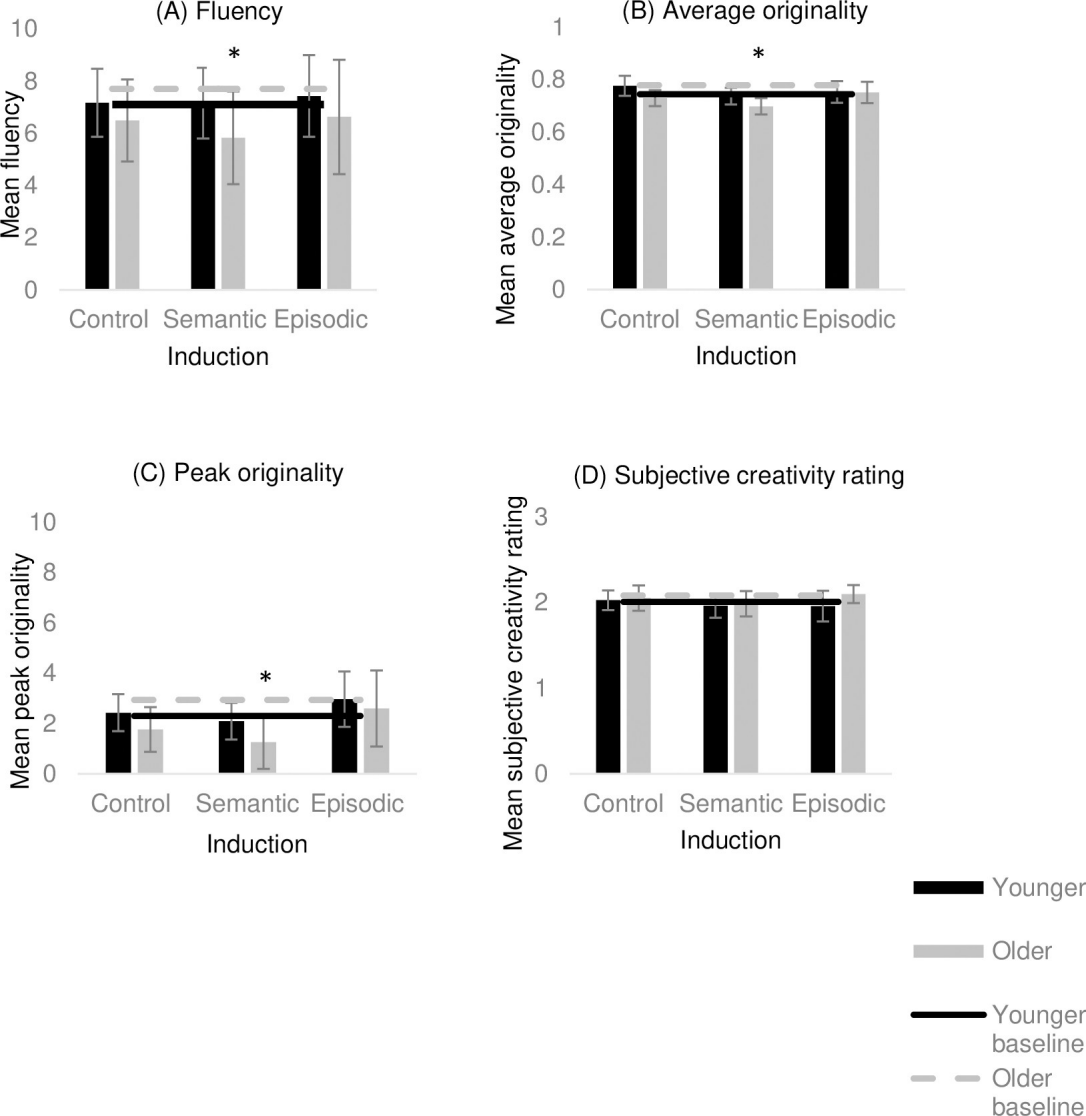
Experiment 1 findings. (A) mean fluency, (B) mean average originality, (C) mean peak originality, and (D) mean subjective creativity rating following each induction for young and older adults. Error bars represent 95% CI. *Note*. * Significantly different from no-induction baseline at Bonferroni corrected *p* value (< .017).

With regard to the three originality measures, there were no significant differences following the episodic (*t*(29) = -.417, *p* = .679, *d* = .09), semantic (*t*(29) = .392, *p* = .698, *d* = -.07) and control (*t*(29) = .392, *p* = .698, *d* = -.07) inductions compared to the no-induction baseline in relation to average originality. This pattern was also true for peak originality (episodic: *t*(29) = -.304, *p* = .763, *d* = 0.19; semantic: *t*(29) = .576, *p* = .569, *d* = .06; control: *t*(29) = -1.21, *p* = .233, *d* = .04) and the subjective creativity ratings (episodic: *t*(29) = .509, *p* = .614, *d* = .17; semantic: *t*(29) = .468, *p* = .643, *d* = .14; control: *t*(29) = -.251, *p* = .804, *d* = .004) (pbonferroni = .017). This indicates that younger adults did not perform significantly better or worse on any measure of the AUT following any of the inductions compared to their no-induction baseline performance (see Fig 2A–2D).

For the older adults, there was a significant difference for fluency following the semantic induction compared to the no-induction baseline (*t*(29) = 4.59, *p* < .001, *d* = .40) such that performance was poorer following the induction. There was no significant difference following the episodic induction (*t*(29) = 1.82, *p* = .079, *d* = .23) or the control induction (*t*(29) = 2.21, *p* = .035, *d* = .25) compared to the no-induction baseline (pbonferroni = .017). These findings suggest a decline in fluency on the AUT in older adults as a result of the semantic induction in comparison to when there is no induction was implemented prior to completing the task (see Fig 2A). With regard to the three originality measures, there was a significant difference for average originality (*t*(29) = 3.84, *p* = .001, *d* = 1) and peak originality (*t*(29) = 3.84, *p* = .001, *d* = .54) following the semantic induction compared to the no-induction baseline such that performance was poorer following the induction.

There was no significant difference for average originality (*t*(29) = 2.08, *p* = .047, *d* = .66) and peak originality (*t*(29) = 2.08, *p* = .047, *d* = .38) between the no induction baseline and following the control induction (pbonferroni = .017). There was also no significant difference on average originality (*t*(29) = 1.00, *p* = .325, *d* = .33) or peak originality (*t*(29) = 1.00, *p* = .325, *d* = .10) following the episodic induction compared to the no-induction baseline. In relation to subjective creativity ratings, there were no significant difference between the no-induction baseline and following the episodic (*t*(29) = -.197, *p* = .845, *d* = .03), semantic (*t*(29) = 1.08, *p* = .289, *d* = .26) and control (*t*(29) = .257, *p* = .799, *d* = .09) inductions (pbonferroni = .017).

Taken together, the above results indicate poorer performance on average originality and peak originality following only the semantic induction compared to the no-induction baseline scores in older adults (see Fig 2B–2D).

### Discussion

Experiment 1 adopted commonly used tasks to assess the two facets of declarative memory for the episodic and semantic induction. The episodic induction focused on personal event construction via an autobiographical memory retrieval task while the semantic induction comprised of a free association task. With regards to induction responsivity, older adults generated more associations than younger adults in the semantic induction, while younger adults generated more internal details than older adults in the episodic induction. These findings fit with well-established patterns in the literature regarding the decline in episodic memory and intact semantic memory in healthy older adults [17].

With regard to episodic memory, the proportion of internal details generated by both younger and older adults in the episodic induction are comparable to those reported in the literature where the same scoring procedure has been adopted for an autobiographical memory-based task [e.g., 16] which suggests that participants engaged with the memory retrieval task as expected for their age. The episodic induction task used in Experiment 1 was similar to tasks used in prior research examining the effects of episodic induction on divergent thinking, in which participants recalled a specific personal memory in response to a cue word [34].

The greater induction responsivity among older adults compared to younger adults after the semantic induction is also an interesting finding, especially as it suggests factors at play beyond having an intact semantic memory system. One possibility is to consider semantic network-based properties that affect the organization and retrieval of memories in the context of ageing. A recent comparison of differences in semantic network properties as a function of ageing using a megastudy approach revealed that while the lexical networks of older adults have less connectivity, they also consistently show longer path lengths than younger adults. Older adults exhibit more interindividual variability than younger adults which gives rise to "more unique lexical representations" [62]. Indeed, analyses of free association networks across the lifespan evidence clear linear increases in vocabulary size with age [63]. Moreover, older people have been shown to consistently draw on their acquired real-world knowledge (as opposed to their specific past experiences) more than younger adults during even simple decision-making [64]. This may explain increased ease of access to semantic information in the semantic induction (free association) task used in this study.

The results, however, revealed no significant differences in creative performance following either the episodic or semantic inductions compared to the control mathematical reasoning induction for both younger and older adults, indicating no specific advantage or disadvantage associated with the declarative memory inductions.

To our knowledge, this experiment is also the first to implement a within-subjects no-induction baseline to assess creative performance within the same paradigm. The results from this analysis differed with respect to age group. Younger adults showed no significant difference on the AUT measures following any of the three induction conditions compared to the no-induction baseline. So, there was no change in the creative performance of younger people after the episodic or semantic inductions compared to a no-induction baseline. Older adults, however, displayed worse performance following the semantic induction compared to the no-induction baseline on fluency, average originality, and peak originality.

The notion of the ‘fan effect’ may aid in the explanation of the deteriorating influence of the semantic induction in older adults in this context despite greater semantic induction responsivity. The ‘fan effect’ refers to the phenomenon that when participants learn a large set of information, the time to retrieve particular information also increases [65]. Extending this notion to the semantic domain, the effect would suggest that those with a richer store of knowledge, such as that demonstrated by the older adults in the current experiment through higher association fluency, would lead to greater interference between competing representations, therefore placing greater demands on control processes [66, but see 67]. It has also been suggested that having a more extensive knowledge store (as is the case with older people) can lead to proneness to interference during semantic retrieval owing to having to choose between several relevant competing representations, which in turn necessitates greater cognitive control [66, 68]. Engagement in the semantic induction free association task could have therefore led to greater demands on control processes, leading to fatigued performance in the subsequent AUT in older adults.

The outcome of the current experiment is contrary to that of Beaty et al. [34], who found that the semantic induction increased the originality of responses in younger adults. Two considerations should be borne in mind when attempting to reconcile these conflicting findings. First and foremost, it is necessary to acknowledge that interpretation of the behavioural evidence of the Beaty et al. study in relation to the semantic induction is limited given that they did not include a no-induction baseline. So, no comparisons could be made between performance following declarative memory inductions and a baseline condition where there was no induction implemented prior to the AUT. As the current experiment demonstrates, only when compared with no-induction baseline performance do older adults show poorer performance following the semantic induction, further highlighting the need for such neutral baseline conditions. Second, Beaty et al. deployed the use of Latent Semantic Analysis (LSA) to measure the originality of responses, where the greater the semantic distance between the AUT object and the use, the greater the originality of the response. Although some scholars have reported the usefulness of adopting LSA when measuring the originality of responses [e.g., 69–71], the use of LSA as a measure of creative quality has been criticised for being more sensitive to the responses following the semantic induction compared to the episodic induction [34] due to the primary use of it being to detect semantic similarities. The current experiment adopted more commonly used methods of scoring originality of responses and additionally included both objective and subjective method of scoring.

A potential limitation of the episodic induction in this experiment concerns the age of the memories retrieved and the possible impact this may have had on the degree of engagement in the episodic retrieval orientation being induced. This is relevant because there is evidence to suggest that the episodic specificity of a memory declines as the memory grows older and have been evidenced in studies using both time-period-based and cue-word-based autographical memory retrieval tasks [72–74]. However, in the current experiment, access to episodic memories was supported by two means: (a) participants were not restricted to retrieve memories from certain lifetime periods when responding to a cue, and (b) they were given the option to receive another cue word if they felt they could not adequately recall an associated memory in detail (based on the retrieval prompts presented to participants that encouraged episodic specificity: e.g., where did it happen?) in response to the cue presented.

In sum, the findings of Experiment 1 indicate that older adults and younger adults demonstrated comparable creative performance and inducing aspects of ‘episodicity’ associated with *personal event-based* memory retrieval, as implemented in this experiment, was not associated with any subsequent advantage on creative performance. The above findings also emphasise the importance of a within-subjects creative performance baseline to be able to accurately evaluate the contribution of such memory inductions (better, worse, or no different from baseline).

## Experiment 2

### Introduction

An increasing number of studies provide evidence that episodic memory not only supports the recollection of the past but also supports the ability to ‘mentally travel’ into the future and consider alternative past experiences (i.e., episodic counterfactual thinking), suggesting that episodic memory contributes largely to imagining or simulating possible future and past experiences [75–77]. While investigating the processes involved in episodic memory that may be contributing to the effects of the ESI, Madore, Addis and Schacter [78] considered the effects of a novel *imagination specificity induction*. Using the experimental recombination paradigm introduced by Addis and colleagues [79], participants were given three details from different events and were asked to verbally create a new event that could happen to them within the next few years that included the three details. They found that both the memory and imagination specificity inductions resulted in a similar increase in the number of internal details on subsequent memory and imagination tasks compared to a control induction. They predicted that like the ESI, the imagination specificity induction would also boost performance on the alternate uses task (p.11), but this hypothesis has not yet been tested.

Experiment 2 employs a similar imagination task for the episodic induction in which participants are presented with a word pair consisting of two unrelated words, and are required to imagine and verbalise a new story involving themselves and the two words (personal imaginative event construction). To maintain comparability between the episodic and semantic inductions with regards to the materials used, an associative distance task was performed as part of the semantic induction. Participants were required to provide an associative pathway, progressively connecting two unrelated words together. Evidence suggests more ‘original’ or highly creative individuals demonstrate shorter associative distances between unrelated word pairs than less ‘original’ individuals [42].

A significant difference is expected in relation to the AUT performance following the episodic induction compared to a semantic induction, control induction and no-induction baseline, such that the episodic induction will lead to better AUT performance. We also predict there will be a significant difference between AUT performance following the semantic and control induction, based on evidence outlined above [42]. It is expected that there will be a significant difference between AUT performance following the episodic and semantic inductions compared to the no-induction baseline for younger and older adults, though the direction of the difference is uncertain, given the findings in Experiment 1.

### Materials and methods

#### Participants

Thirty younger adults (18–30 years) and thirty older adults (60–80 years) participated in the experiment. Five participants were excluded due to not engaging in the inductions as instructed. Therefore, the final sample consisted of 29 younger adults (*M* = 22.59, *SD* = 3.51; 18 women, 11 men) and 26 older adults (*M* = 73.85, *SD* = 4.76; 20 women, 6 men). The participants were recruited using the same eligibility criterion and screening processes as in Experiment 1. All participants provided written informed consent prior to participation. There was no significant difference in IQ between younger (*M* = 104.79, *SD* = 9.02) and older adults (*M* = 101.31, *SD* = 8.63) (*t*(53) = 1.46, *p* = .150, *d* = .38). For the consistency of analysis across the three experiments, IQ was included in the analysis as a covariate (i.e., ANCOVAs were performed).

#### Tasks

*1*. *Episodic induction*. A modified version of the Scene Construction Task by Hassabis and colleagues [77] was adopted to evoke episodic retrieval in the episodic induction. In the original Scene Construction Task, participants are required to imagine a novel scenario (e.g., “Imagine you’re lying on a white sandy beach in a beautiful tropical bay”). They are explicitly instructed not to recall an actual memory, but to create something new. In order to ensure comparability between the two declarative memory inductions in the present experiment, participants were presented with word-pairs taken from Gianotti et al. [55] and were instructed to imagine a novel story involving themselves and the word-pair. Instructions were taken from Hassabis et al. [80] to closely replicate the imagination task and were only modified slightly to change the word ‘scene’ to ‘story’. Participants were instructed to be as vivid as possible in their description and include a series of events in their story including episodic details. A detailed example, rich in episodicity, was provided. Word-pairs were presented in a random order to each participant. The number of word-pairs for each participant varied in order to complete 5 minutes induction length (range 1–5). When response to one word-pair had naturally elapsed, the next word-pair was presented. Responses were audio recorded and transcribed.

#### Episodic induction scoring

As in Experiment 1, episodic induction responsivity was scored for internal and external details using the Text Segmentation and Categorisation scoring technique from Levine and colleagues [16]. Two raters scored 12 responses (22% of all responses) and obtained high interrater reliability (Cronbach’s α = .97). Raters were blind to which age group the story description came from. There was a significant difference in the number of word-pairs adopted in the episodic induction by younger (*M* = 2.52, *SD* = 1.12) and older adults (*M* = 1.62, *SD* = .80) (*t*(53) = 3.39, *p* < .01, *d* = 1.12). This was not considered an issue as the length of induction remained at 5 minutes for all participants. Given that it was necessary to control for the length of time participants engaged in the inductions, it was expected that there may be differences in the number of cue words required to reach the 5-minute time limit.

*2*. *Semantic induction*. A modified version of the task used in Rossman and Fink [42], in which participants judge the relatedness of word pairs, was adopted for the semantic induction. In the present experiment participants were presented with unrelated word pairs (e.g., *letter-family*) taken from Gianotti et al. [55] and were required to provide a link of associated words that connect the two words of the pair together (e.g., *letter–* ***homesick*** *–family)*. Participants were explicitly instructed that there are no fixed number of words they must provide, the number of words can range from 1 up to however many they think are necessary to connect to the second word. The duration of induction was 5 minutes; therefore the number of word pairs presented to each participant varied in order to complete the 5 minutes induction phase (range 6–14). Word pairs were presented in a random order to participants.

#### Semantic induction scoring

For each participant, the number of words used to connect each pair (path length) was calculated (e.g., *letter–* ***homesick*** *–family*, this example uses 1 connecting word). An average path length was then calculated for each participant. There was no significant difference in the number of word-pairs adopted in the semantic induction by younger (*M* = 10.62, *SD* = 3.22) and older adults (*M* = 11.12, *SD* = 3.23) (*t*(53) = -.568, *p* = .573, *d* = .15).

*3*. *Control induction*. The same non-verbal math sequence task from Experiment 1 was used as a control induction consisting of 5 trials for all participants to complete the 5-minute induction phase.

*4*. *Alternate uses task*. The same task of divergent thinking was employed as in Experiment 1 and was administered and scored in the same way. For the subjective creativity ratings, the raters showed good interrater reliability (Cronbach’s α = .79) In Experiment 2, objects included were *knife*, *key*, *brick*, and *shoe*.

#### Experimental design and procedure

Experiment 2 followed the same procedure as Experiment 1.

### Results

#### Induction responsivity

**Fig 3:**
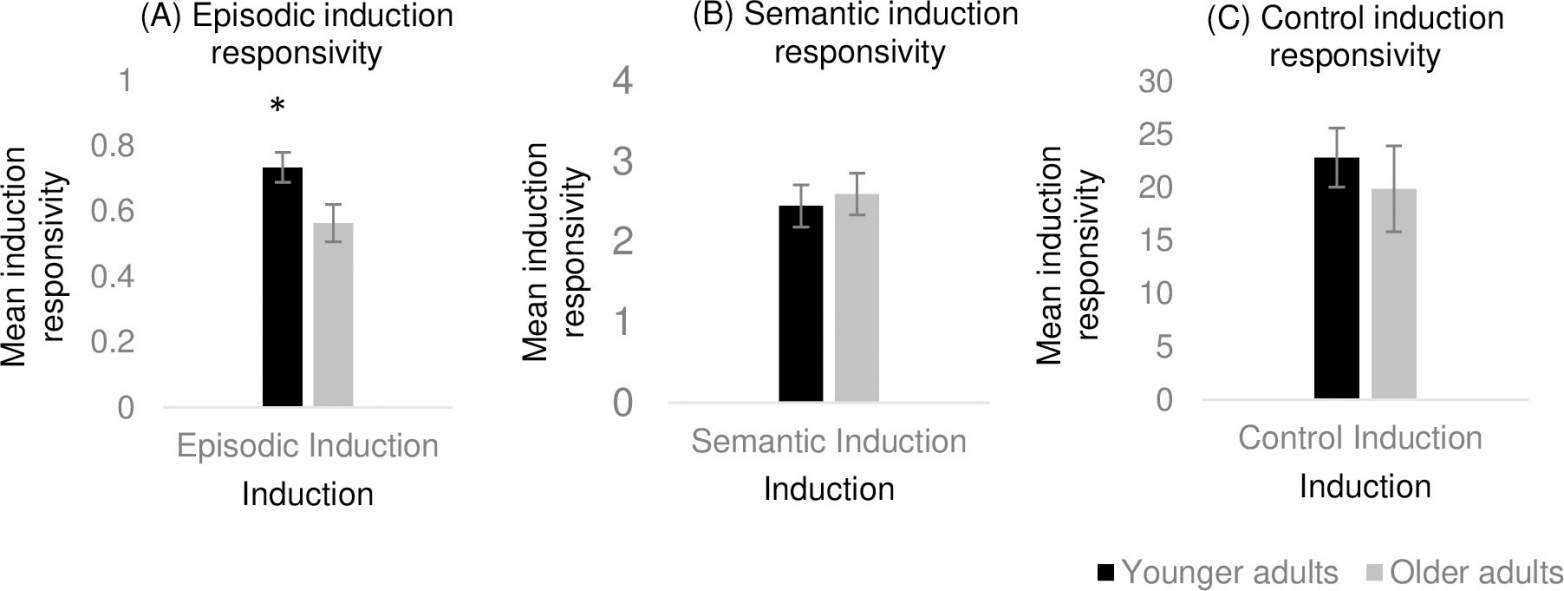
Experiment 2 findings. Mean induction responsivity score in each induction task for young and older adults. Error bars represent 95% CI. *Note*. * Significant difference between younger and older adults (*p <* .05).

To investigate whether younger and older adults differed in their behavioural responses during the induction procedures, a series of one-way ANCOVAs were conducted. There was a significant difference between younger (*M* = .73, *SD* = .12) and older adults (*M* = .56, *SD* = .14) on the episodic induction responsivity score (*F*(1, 53) = 23.46, *p* < .001, η^2^ = .31), such that younger adults generated significantly more internal details than older adults (Fig 3A). There was no significant difference between younger and older adults in semantic induction responsivity (*F*(1, 53) = 5.65, *p* = .455, η^2^ = .01) (Fig 3B) and control induction responsivity (*F*(1, 53) = 1.57, *p* = .216, η^2^ = .02) (Fig 3C).

#### Order-based differences

As in Experiment 1, a series of one-way ANCOVAs were conducted prior to the main statistical analyses to check for induction/object order effects. No significant differences were found for fluency (*F*(2, 52) = .150, *p* = .887, η^2^ = .004), average originality (*F*(2, 52) = .448, *p* = .641, η^2^ = .02), peak originality (*F*(2, 52) = .149, *p* = .862, η^2^ = .01), and subjective creativity ratings (*F*(2, 52) = .264, *p* = .769, η^2^ = .01) as a function of induction order. There were also no significant differences as a function of the AUT order on fluency (*F*(3, 51) = 2.10, *p* = .112, η^2^ = .11), average originality (*F*(3, 51) = .653, *p* = .585, η^2^ = .04), peak originality (*F*(3, 51) = 2.24, *p* = .094, η^2^ = .12), and subjective creativity ratings (*F*(3, 51) = .289, *p* = .833, η^2^ = .02). This confirms that there was no effect of the order in which participants received the induction, nor any effect of the order in which participants received the AUT objects after each induction on the four features of creative thinking.

#### Differences following episodic, semantic and control inductions

**Fig 4:**
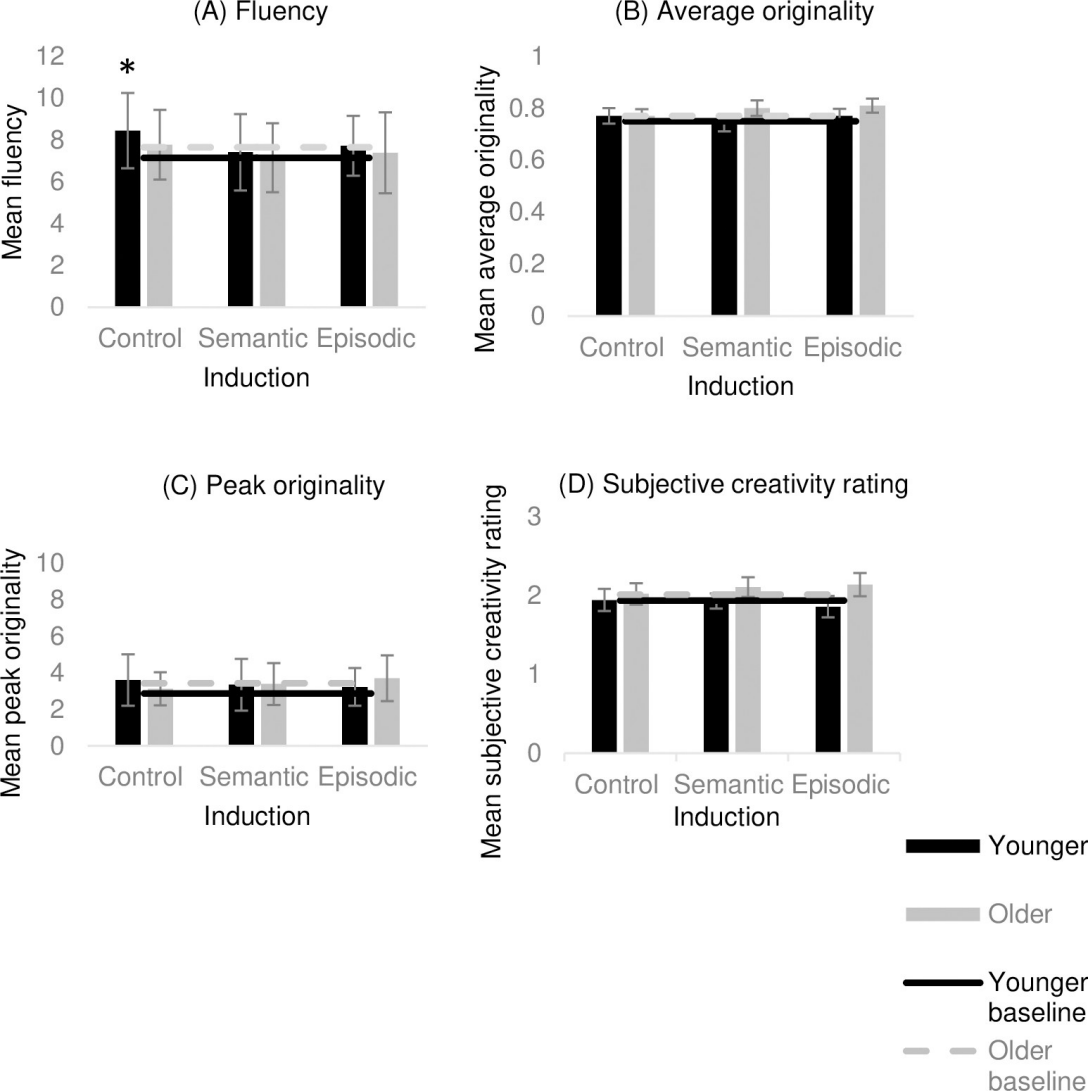
Experiment 2 findings. (A) mean fluency, (B) mean average originality, (C) mean peak originality, and (D) mean subjective creativity rating following each induction for young and older adults. Error bars represent 95% CI. * Significantly different from no-induction baseline at Bonferroni corrected *p* value (< .017).

A series of 3 (Induction: episodic, semantic and control) x 2 (Age: younger vs older) ANCOVAs were conducted in relation to all four AUT measures (see Table 2 for the descriptive findings). With regard to fluency, there was no main effect of induction (*F*(2, 104) = .429 *p* = .653, η_p_^2^ = .008), age (*F*(1,52) = .007, *p* = .932, η_p_^2^ < .000), nor age × induction interaction (*F*(2, 104) = .169, *p* = .844, η_p_^2^ = .003) (see Fig 4A).

**Table 2:** Means (M) and standard deviations (SD) for the alternate uses task (AUT) measures in Experiment 2.

	No-induction baseline		Episodic induction		Semantic induction		Control induction	
	Younger adults	Older adults	Younger adults	Older adults	Younger adults	Older adults	Younger adults	Older adults
AUT measures	*M (SD)*	*M (SD)*	*M (SD)*	*M (SD)*	*M (SD)*	*M (SD)*	*M (SD)*	*M (SD)*
Fluency	7.13 (5.27)	7.65 (5.01)	7.72 (3.86)	7.38 (5.51)	7.41 (4.95)	7.15 (4.71)	8.44 (4.79)	7.77 (4.47)
Average originality	.75 (.09)	.77 (.08)	.77 (.07)	.81 (.07)	.74 (.08)	.80 (.07)	.77 (.08)	.77 (.07)
Peak originality	2.86 (3.71)	3.42 (3.42)	3.21 (2.79)	3.69 (3.52)	3.34 (3.83)	3.38 (3.16)	3.59 (3.77)	3.11 (2.36)
Subjective creativity ratings	1.93 (.31)	2.01 (.38)	1.86 (.36)	2.14 (.37)	1.93 (.25)	2.11 (.31)	1.94 (.37)	2.02 (.33)

With regard to the three originality measures, there was no main effect of induction in relation to average originality (*F*(2, 104) = 1.81, *p* = .168, η_p_^2^ = .034), peak originality (*F*(2, 104) = .915, *p* = .404, η_p_^2^ = .017), and subjective creativity ratings (*F*(2, 114) = .928, *p* = .398, η_p_^2^ = .016). There was, however, a significant main effect of age in relation to average originality (*F*(1, 52) = 8.04, *p* = .007, η_p_^2^ = .113), and subjective creativity ratings (*F*(1,52) = 17.52, *p* < .001, η_p_^2^ = .252), such that older adults performed better than younger adults across all induction conditions on these measures. There was no main effect of age in relation to peak originality (*F*(1,52) = .228, *p* = .635, η_p_^2^ = .004). Finally, there was no significant age × induction interaction in relation to either average originality (*F*(2, 104) = 2.57, *p* = .081, η_p_^2^ = .047), peak originality (*F*(2, 104) = .741, *p* = .479, η_p_^2^ = .014), or subjective creativity ratings (*F*(2, 104) = .836, *p* = .437, η_p_^2^ = .016) (see Fig 4B–4D).

In sum, older adults demonstrated greater average originality and subjective creativity than younger adults and none of the inductions had a significant impact on any measured aspect of divergent thinking.

#### Analyses in relation to the no-induction baseline

A series of one-way ANCOVAs were carried out to assess whether younger and older adults differed on the four dependent measures of the AUT in relation to their no-induction baseline scores. There was no significant difference between younger adults’ and older adults’ baseline fluency (*F*(1, 53) *=* .135, *p* = .714, *d* = -.09), baseline average originality (*F*(1, 53) *=* .459, *p* = .501, *d* = -0.17), baseline peak originality (*F*(1, 53) *=* .337, *p* = .564, *d* = .17), and baseline subjective creativity rating (*F*(1,53) = .677 *p* = .414, *d* = .26). This suggests that both younger adults and older adults performed comparably on the AUT when there is no induction implemented prior to completing the task.

To assess differences on AUT performance following each induction compared to no-induction baseline, a series of paired-samples t-tests were conducted. For younger adults there was no significant difference for fluency following the semantic induction (*t*(28) = -.538, *p* = .298, *d* = .04) and episodic induction (*t*(28) = -.956, *p* = .174, *d* = .11) compared to the no-induction baseline. There was, however, a significant difference for fluency following the control induction (*t*(28) = -3.32, *p* = .001, *d* = .25), such that the younger adults produced significantly more uses following the control math induction compared to the no-induction baseline (pbonferroni = .017) (see Fig 4A). With regard to the three originality measures, there was no significant difference following the episodic induction (*t*(28) = -.731, *p* = .235, *d* = .22), semantic induction (*t*(28) = .573, *p* = .286, *d* = .11), and control induction (*t*(28) = -.591, *p* = .280, *d* = .22) compared to the no-induction baseline in relation to average originality. This pattern was the same for peak originality (episodic: *t*(28) = -.697, *p* = .491, *d* = .09, semantic: *t*(28) = -.980, *p* = .336, *d* = .13, control: (*t*(28) = -2.28, *p* = .030, *d* = 0.18)) and subjective creativity ratings (episodic: *t*(28) = .942, *p* = .354, *d* = .23) semantic: (*t*(28) = .004, *p* = .996, *d* = .01), control: (*t*(28) = -.120, *p* = .905, *d* = .03)) (pbonferroni = .017) (see Fig 4B–4D). In sum, compared to a no-induction baseline, only the control math induction was associated with higher fluency in younger adults.

In older adults, there was no significant difference for fluency following the semantic induction (*t*(25) = 1.09, *p* = .143, *d* = .09), episodic induction (*t*(25) = .357, *p* = .362, *d* = .05), and control induction (*t*(25) = -.167, *p* = .434, *d* = .02) compared to the no-induction baseline (Bonferroni corrected for multiple comparisons; adjusted *p* value = .017) (see Fig 4A). With regard to the three measures of originality, there were no significant differences following the episodic (*t*(25) = -2.24 *p* = .304, *d* = .05), semantic (*t*(25) = -1.678, *p* = .106, *d* = .03), and control induction, (*t*(25) = -.150, *p* = .441, *d* = .00) compared to the no-induction baseline in relation to average originality. This pattern was the same for peak originality (episodic: *t*(25) = -.609 *p* = .548, *d* = 08, semantic: *t*(25) = .091, *p* = .928, *d* = .01, control: *t*(25) = .610, *p* = .548, *d* = .08), and subjective creativity ratings (episodic: *t*(29*)* = -1.19, *p* = .245, *d* = .34, semantic: *t*(29) = -1.04, *p* = .309, *d* = .26, control: *t*(29) = -.109, *p* = .914, *d* = .03). So, older adults did not perform significantly better or worse on the AUT following any of the inductions compared to their no-induction baseline scores on any measures of originality (pbonferroni = .017) (see Fig 4B–4D). In sum, the findings indicate that, compared to a no-induction baseline, the performance of the older adults on the AUT was unaffected by the inductions.

### Discussion

Experiment 2 tested whether an imagination-based episodic induction and a comparable semantic induction would improve creative performance compared to a control math induction and no-induction baseline. As in Experiment 1, the proportion of internal details generated by both younger and older adults in the episodic induction were similar to those reported in the literature (i.e., higher proportions in younger adults) where the same scoring procedure has been adopted to score a task requiring the generation of novel future events [81]. This suggests that participants engaged with the induction as expected for their age.

The results revealed a significant main effect of age in relation to originality (on two measures–average originality and subjective creativity ratings), such that older adults outperformed younger adults regardless of induction conditions. This finding will be discussed in depth in the General Discussion. Just as in Experiment 1, the results revealed no significant difference in creative performance following either the episodic or semantic induction compared to the control mathematical reasoning induction for both younger and older adults. This points to no specific advantages or disadvantages associated with either of the inductions. In relation to the no-induction baseline the findings, once again, differed with respect to age. There was no significant difference on any of the AUT measures following the inductions compared to the no-induction baseline for older adults, indicating that the inductions did not improve or worsen performance for older adults. The younger adults, however, showed an unexpected significant improvement in fluency following the control induction compared to the no-induction baseline.

It is difficult to interpret these findings as the control induction consisted of a non-declarative memory task involving mathematical reasoning. Investigations into the cognitive components of math reasoning skills have provided evidence for the role of semantic fact retrieval as significantly predicting math calculations and math reasoning [82]. The nature of the task is also such that it could induce certain executive function processes that may have had a significant bearing on creative performance [60, 83, 84]. For example, the process of *updating* (monitoring of incoming information and the revision of content in the focus of one’s attention) is a key component of working memory [85], which has been found to significantly predict creative performance [60, also see 86]. Alternatively, it is possible that this finding represents a statistical oddball. As no predictions were put forward in relation to the control induction, these findings are not discussed further.

A potential concern with the current paradigm pertains to the semantic induction implemented. In aiming to maintain comparability between the task characteristics, a modified version of the Rossman and Fink [43] task was adopted, where participants are asked to provide an associative pathway in order to link the two words of the word-pair together. Due to the nature of the adaptation of the original task, it is possible that participants imagined events or remembered past events which could allow them to create a link between the word-pairs. Therefore, it cannot be ruled out that some participants may have engaged in episodic processes during the semantic induction. This might have contributed to the lack of significant findings in relation to the semantic induction in Experiment 2. Evidence regarding the interdependence of the two memory systems [e.g., 87–89] implies that episodic and semantic induction procedures will inevitably overlap to some extent with regard to their operations. Nevertheless, it is important to note that all the experiments reported thus far devised novel induction procedures which aimed to target and induce more episodic-related processes during the episodic inductions and more semantic memory-related processes during the semantic inductions.

In sum, the findings of Experiment 2 indicate that older adults outperformed younger adults on some aspects of divergent thinking and that inducing aspects of ‘episodicity’ concerning the construction of personal imagined events, as implemented in this experiment, was not associated with any subsequent advantage on creative performance.

## Experiment 3

### Introduction

Experiments 1 and 2 were designed to tap into different aspects of episodicity including personal event-based memory retrieval (personal past event construction) and construction of imagined personal events (personal imagined event construction), yet did not yield similar findings to those previously associated with episodic memory induction and divergent thinking [7, 8]. The aim of Experiment 3 was therefore to use the same episodic induction procedure (ESI) as in the first study to showcase such effects [7] to evaluate the effect of the same on divergent creative thinking and to assess whether previous findings may perhaps be specific to the type of memory induction implemented.

This induction procedure was incorporated into the general format of the paradigms used in the previous studies as follows. First, in addition to using the same ESI protocol, the present experiment includes a no-induction baseline. Second, for consistency with Experiments 1 and 2, the ‘impressions control induction’ developed by Madore and colleagues [7], was implemented with some modifications which entailed the replacement of some questions with others that were designed to induce semantic memory operations. Finally, the original study by Madore, Jing and Schacter [8] assessed AUT measures of fluency, flexibility, elaboration, and originality (as measured by subjective ratings) and found significant effects of the ESI on fluency and flexibility but no significant effect of the ESI on originality. The present experiment will assess fluency, flexibility, and originality. The latter will be scored via subjective creativity ratings, as in the original study by Madore and colleagues [8], as well as via the objective scoring methods of creativity used in Experiments 1 and 2 (average originality and peak originality measures).

Given that the ESI was found to boost performance on the AUT and various other cognitive tasks, such as means-end problem solving [18], in comparison to an impressions control induction and a neutral math control induction, we predicted that there would be a significant difference between the AUT performance following the ESI compared to a no-induction baseline and semantic induction. Contrary to the findings of Madore and colleagues [8], we predicted a significant difference between younger and older adults on the fluency and average originality measures of the AUT based on the pattern of findings from Experiments 2, such that older adults will generate more uses and more original uses than younger adults.

### Materials and methods

#### Participants

A total of 36 younger adults (18–30 years) and 31 older adults (60–80 years) participated in the experiment. One participant was excluded from the analysis due to disengagement in the induction condition, resulting in a final sample of 36 younger adults (*M* = 19.8, *SD* = 2.91; 30 women, 5 men and 1 other) and 30 older adults (*M* = 70.97, *SD* = 5.39; 23 women, 7 men). The participants were recruited and screened using the same procedures as Experiments 1 and 2. All participants provided written informed consent prior to participation. There was a significant difference in IQ between younger (*M* = 107.11, *SD* = 7.31) and older adults (*M* = 100.90, *SD* = 8.73) (*t*(64) = 3.15, *p* = .003, *d* = .84). Therefore, IQ was entered as a covariate in the subsequent analyses (i.e., ANCOVAs were performed).

#### Tasks

*1*. *Episodic Specificity Induction (ESI)*. The ESI [7, 8] is based on the Cognitive Interview (CI), commonly used in eyewitness recall and is designed to boost the number of accurate details [90, 91]. In the ESI, participants view one of two short videos of a man and woman carrying out tasks in a kitchen setting. They are then told that they are the experts on the video and are questioned about the video using probes from the CI. Participants are guided through three mental imagery problems, the surroundings, the actions, and the people in the video. They are instructed to close their eyes and picture everything they remember. They are then asked to verbalise everything they remember in as much detail as possible. Furthermore, they are probed with open-ended questions to elaborate on details they had mentioned. The ESI was implemented using the same protocol as in previous studies [7, 8] and lasted a maximum of 10 minutes to allow sufficient time to recall all three elements.

*2*. *Semantic induction*. This was a modified version of the Impressions Control Induction used in previous studies [7] whereby participants were asked a series of general questions regarding the video. Questions that were likely to trigger autobiographical memories (e.g., Did the video remind you of anything from your own life?) were either removed or exchanged for questions requiring semantic operations (e.g., Was the video similar to something else you know or have seen?). Three questions in total were replaced.

*3*. *Alternate Uses Task (AUT)*. The same divergent thinking task was used as in Experiments 1 and 2. To closely follow prior experiments in relation to the divergent thinking task [7, 8], in Experiment 3, five trials of the AUT were completed after receiving each induction, resulting in a total of 15 objects for which uses were generated [8, taken from the supplementary material]. Madore and colleagues [7] provided participants with a practice AUT trial before completing the task (resulting in 6 trials in total after each induction). As participants were required to record responses electronically, the practice trial was to allow participants to familiarise themselves with the method [7, see 8 for *think aloud* procedure used]. This was not deemed necessary in the current experiment as participants reported their responses out aloud and responses were recorded by the experimenter. Additionally, evidence suggesting a linear reduction of fluency over 5 trials and severe reduction at trial 6 [57] advocates the rationale for the elimination of a practice trial which may otherwise result in detrimental performance in the final experimental trial based on the above pattern.

Experiments 1 and 2 consisted of a single trial of AUT after each induction, whereas Experiment 3 consisted of 5 trials of AUT in the no-induction baseline condition and following each induction. As such, an average of the 5 trials was calculated to obtain scores of fluency, average originality, peak originality, and subjective creativity ratings across all conditions. For the subjective creativity ratings, raters revealed good interrater reliability (Cronbach’s α = .76). In addition to measuring the above indices of the AUT as in Experiments 1 and 2, Experiment 3 included the measure of *flexibility*.

Flexibility refers to uses that are clustered into distinct categories [7]. For example, using a coin as a pendant or a ring would both come under the category of *jewellery*. The flexibility score represents the number of distinct categories a participant has generated with at least one use within the category (e.g., at least one use within the *jewellery* category). The number of categories generated for each AUT trial was averaged across the 5 trials to obtain a flexibility score following each induction and the no-induction baseline.

#### Experimental design and procedure

Participants attended three one-to-one lab sessions for testing spaced at least one week apart and took part in all three conditions: no-induction baseline, ESI, and semantic induction. The number of sessions attended was different to Experiment 1 and 2 in order to follow the procedure of past studies [7, 8]. All participants took part in the no-induction baseline condition in session 1, in which they completed the AUT, executed the same way as in Experiments 1 and 2. They then completed the 9-item short Form A of the Raven’s Standard Progressive Matrices (RSPM) [52]. In sessions 2 and 3 participants (a) watched one of two short videos of a man and woman in a kitchen setting, (b) received either the episodic specificity or semantic induction, (c) completed the AUT immediately after the induction. The order in which the participants received the episodic and semantic inductions, either session 2 or 3, was counterbalanced, as were the videos watched in each of the inductions and the order in which the videos were presented. The AUT trials following each of the inductions was counterbalanced. Participants were randomly assigned to which induction order they received. Counterbalancing was executed in such a way that there was a comparable number of younger and older adults in each induction/AUT trial order (n = 16–17 per induction order group). All sessions lasted approximately 45 minutes.

### Results

The analyses for Experiment 3 followed the same procedures as in Experiment 1 and 2.

#### Order-based differences

A series of one-way ANCOVAs were conducted, which revealed no significant differences as a function of induction order on fluency (*F*(3, 62) = 1.33, *p* = .270, η^2^ = .06), average originality (*F*(3, 62) = 2.09, *p* = .110, η^2^ = .09), peak originality (*F* (3, 62) = 1.55, *p* = .210, η^2^ = .06), subjective creativity ratings (*F(*3, 62) = .581, *p* = .630, η^2^ = .03), and flexibility (*F*(3, 62) = 1.26, *p* = .296, η^2^ = .06). There was also no significant difference on any of the 5 measures as a function of the AUT order on fluency (*F*(14, 51) = 1.05, *p* = .426, η^2^ = .22), average originality (*F*(14, 51) = 1.37, *p* = .202, η^2^ = .27), peak originality (*F*(14, 51) = 1.21, *p* = .299, η^2^ = .25), subjective creativity (*F*(14, 51) = .993, *p* = .474, η^2^ = .21), and flexibility (*F*(14, 51) = .966, *p* = .500, η^2^ = .21). This confirms that there was no effect of the order in which participants received the induction and no effect of the order in which participants received the AUT objects after each induction on the 5 features of creative thinking.

#### Differences following episodic, semantic and control inductions

**Fig 5:**
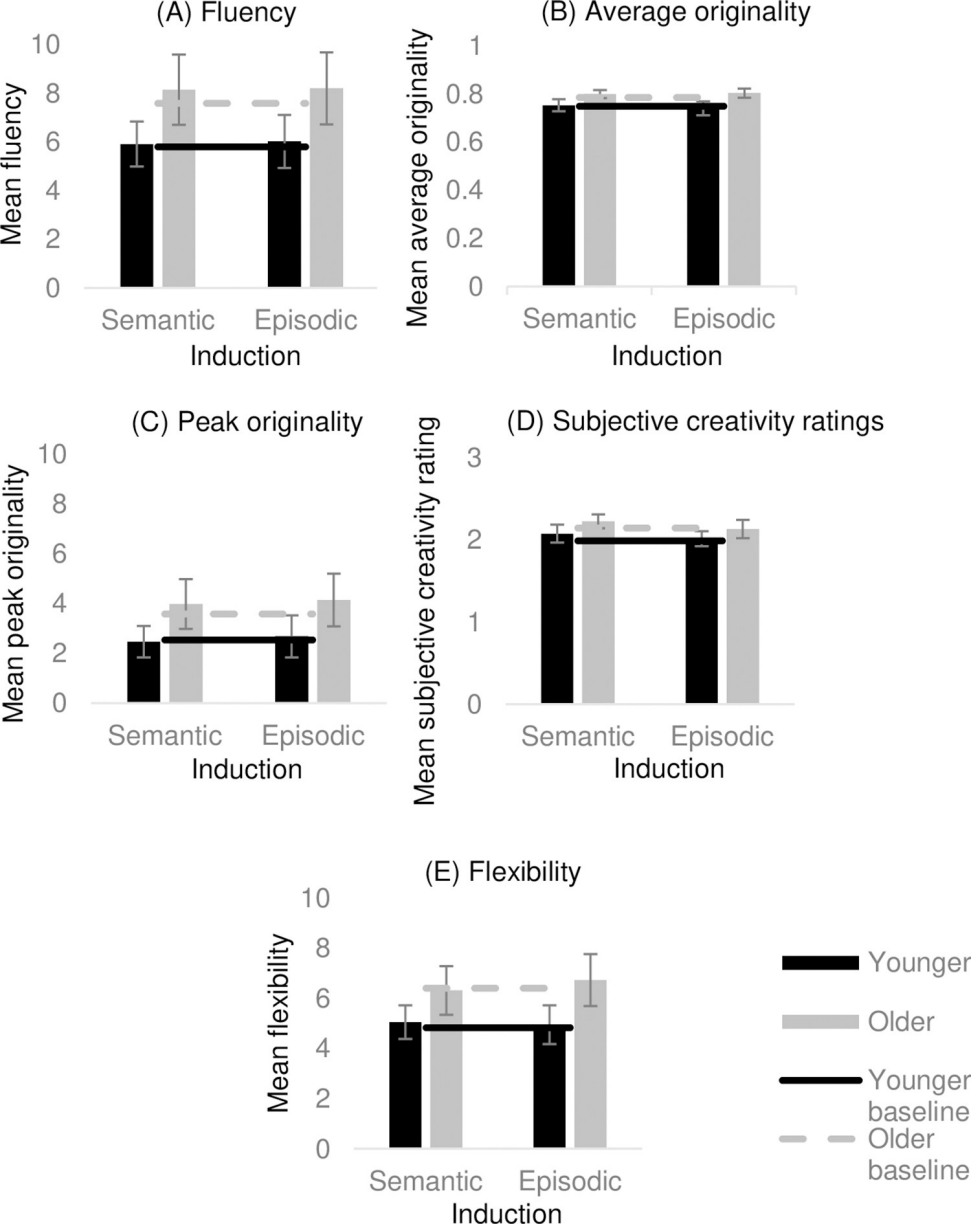
Experiment 3 findings. (A) mean fluency, (B) mean originality, (C) mean peak originality, (D) mean subjective creativity rating, and (E) mean flexibility following each induction for young and older adults. Error bars represent 95%. There was a significant main effect of age on all five measured aspects of the AUT, such that older adults performed better than younger adults.

A series of 2 (Induction: ESI and semantic) × 2 (Age: younger vs older) ANCOVAs were conducted in relation to all five AUT measures to evaluate whether the episodic and semantic inductions had an impact on the creativity measures for both younger and older adults in comparison to the control math induction (see Table 3 for descriptive findings). With regard to fluency, there was no main effect of induction (*F*(1, 63) = .498, *p* = .483, η_p_^2^ = .008), but there was a significant main effect of age (*F*(1, 63) = 6.79, *p* = .011, η_p_^2^ = .097) such that older adults generated more uses than younger adults regardless of the induction condition (Fig 5A). There was no age × induction interaction (*F*(1, 63) = .019, *p* = .892, η_p_^2^ = .019).

**Table 3:** Means (M) and standard deviations (SD) for the alternate uses task (AUT) measures in Experiment 3.

	No-induction baseline		Episodic specificity induction		Semantic induction	
	Younger adults	Older adults	Younger adults	Older adults	Younger adults	Older adults
AUT measures	*M (SD)*	*M (SD)*	*M (SD)*	*M (SD)*	*M (SD)*	*M (SD)*
Fluency	5.81 (3.14)	7.59 (3.26)	6.03 (3.21)	8.21 (3.96)	5.92 (2.27)	8.16 (3.88)
Average originality	.75 (.07)	.79 (.06)	.74 (.08)	.80 (.05)	.75 (.07)	.79 (.04)
Peak originality	2.53 (2.31)	3.58 (2.26)	2.68 (2.5)	4.14 (2.83)	2.46 (1.86)	3.98 (2.67)
Subjective creativity ratings	1.99 (.27)	2.15 (.26)	2.02 (.27)	2.14 (.29)	2.08 (.32)	2.23 (.23)
Flexibility	4.83 (2.12)	6.41 (2.58)	4.95 (2.29)	6.73 (2.75)	5.1 (1.99)	6.31 (2.37)

With regard to the three originality measures, there was a significant main effect of age in relation to average originality (*F*(1, 63) = 10.86, *p* = .002, η_p_^2^ = .15), peak originality (*F*(1, 63) = 6.78, *p* = .012, η_p_^2^ = .096), and subjective creativity ratings (*F*(1, 63) = 6.29, *p* = .015, η_p_^2^ = .091). The main effect of age was such that older adults exhibited higher originality than younger adults following both inductions in relation to all measures of originality (Fig 5B–5D). There were neither any significant main effects of induction nor any significant age × induction interaction effects in relation to average originality (induction: *F*(1, 63) = .087, *p* = .769, η_p_^2^ = .001, age x induction: *F*(1, 63) = 1.18, *p* = .281, η_p_^2^ = .018), peak originality (induction: *F*(1, 63) = .000, *p* = .992, η_p_^2^ < .000, age x induction: *F*(1, 63) = .015, *p* = .903, η_p_^2^ < .000), or subjective creativity ratings (induction: *F*(1, 63) = .076, *p* = .783, η_p_^2^ = .001, age x induction: *F*(1, 63) = .202, *p* = .655, η_p_^2^ = .003).

With regard to flexibility, there was no main effect of induction (*F*(1, 63) = .1.73, *p* = .193, η_p_^2^ = .027) and no age × induction interaction (*F*(1, 63) = 2.73, *p* = .103, η_p_^2^ = .042). A significant main effect of age (*F*(1, 63) = 7.27, *p* = .009, η_p_^2^ = .103) was found such that older adults showed higher flexibility in idea generation than younger adults by producing significantly more categories of uses regardless of induction condition (Fig 5E).

In sum, none of the inductions had a significant impact on any measures of divergent thinking. Older adults showed better performance than the younger adults on all measured aspects of divergent thinking: fluency, flexibility, peak originality, average originality, and subjective creativity ratings.

#### Analyses in relation to the no-induction baseline

Among younger adults, there were no significant differences on any measure of divergent thinking following the ESI relative to the no-induction baseline on fluency (*t*(35) = -.557, *p* = .581, *d* = .07), average originality (*t*(35) = .643, *p* = .542, *d* = .12), peak originality (*t*(35) = -.542, *p* = .591, *d* = .06), subjective creativity ratings: (*t*(35) = -.458, *p* = .650, *d* = .11), or flexibility (*t*(35) = -.373, *p* = .712, *d* = .06). Similarly, there were also no significant differences on any measures of divergent thinking following semantic induction relative to the no-induction baseline on fluency (*t*(35) = -.355, *p* = .724, *d* = .04), average originality (*t*(35) = -.373, *p* = .711, *d* = .00), peak originality (*t*(35) = .292, *p* = .772, *d* = .03), subjective creativity ratings (*t*(35) = -1.13, *p* = .265, *d* = .33), or flexibility (*t*(35) = -.835, *p* = .410, *d* = .13) (pbonferroni = .025).

For older adults as well, there were no significant differences on any measure of divergent thinking following the ESI relative to the no-induction baseline on fluency (*t*(29) = -1.86, *p* = .103, *d* = .16), average originality (*t*(29) = -1.40, *p* = .170, *d* = .16), peak originality (*t*(29) = -1.718, *p* = .096, *d* = .25), subjective creativity ratings (*t*(29) = .150, *p* = .882, *d* = .04), or flexibility (*t*(29) = -.841, *p* = .407, *d* = .12). Similarly, there were also no significant differences on any measures of divergent thinking following semantic inductions relative to the no-induction baseline on fluency (*t*(29) = -1.86, *p* = .103, *d* = .17), average originality (*t*(29) = -1.15, *p* = .259, *d* = .00), peak originality (*t*(29) = -1.405, *p* = .171, *d* = .18), subjective creativity ratings (*t*(29) = -1.32, *p* = .196, *d* = .31), or flexibility (*t*(29) = .234, *p* = .817, *d* = .04) (pbonferroni = .025). In sum, this indicates that neither the younger adults nor older adults performed better or worse following the ESI or semantic induction on any of the measure of the AUT when compared to a no-induction baseline.

To test for the consistency of age-related differences in the absence of experimental or control inductions, a series of one-way ANCOVAs were conducted on the five AUT measures between the younger and older adults in the no-induction baseline condition. There was a significant difference between younger and older adults’ performance on fluency (*F*(1,64) = 5.15, *p* = .027, *d* = 0.57), average originality (*F*(1,64) = 4.38, *p* = .040, *d* = 0.49), flexibility (*F*(1,64) = 7.45, *p* = .008, *d* = 0.74) and subjective creativity ratings (*F*(1,64) = 5.47, *p* = .022, *d* = 0.57) in the no-induction baseline condition. There was no significant difference between younger and older adults on peak originality (*F*(1,64) = 3.47, *p* = .067, *d* = 0.45) in the no-induction baseline condition. This indicates that older adults performed better than younger adults on four out of the five measures of divergent thinking at baseline when there is no form of induction implemented.

### Discussion

The main aim of Experiment 3 was to evaluate whether adopting the ESI prior to a divergent thinking task would result in similar findings to the first study investigating the contribution of episodic memory processes on divergent thinking using the episodic specificity induction (ESI) [7, 8]. The original study reported that ESI had a beneficial impact on fluency and flexibility during divergent thinking but not on subjective creativity ratings [7].

There were no significant findings in relation to the ESI or the semantic induction on any of the five measures of divergent thinking in the current experiment. The results therefore do not support the findings of Madore et al. [7], which identified a boosting role of episodic memory operations in fluency and flexibility during divergent thinking. Moreover, contrary to the findings of Madore, Jing and Schacter [8], who showed that younger and older adults performed comparably on all measures of the AUT following both the ESI and control induction, the current experiment revealed a consistent effect of age, such that older adults outperformed younger adults across all measures of divergent thinking on the AUT regardless of whether or not they had undergone a prior declarative memory induction. Given the extent of similarity in the way in which the AUT was executed in the present study and that by Madore et al. [8] (i.e., ample time to allow responses and providing responses out aloud), the disparate age-related findings are of particular interest and will be examined further in the General Discussion. These age-related findings contribute to the wider literature on the effect of age on creativity, the findings of which are decidedly mixed as they suggest either preserved performance by older adults [11–13, 92] or reduced performance compared to younger adults [93, 94] in divergent thinking.

In sum, the findings of Experiment 3 indicate that older adults outperformed younger adults on all measured aspects of divergent thinking, and that inducing aspects of ‘episodicity’ via the ESI in this experiment was not associated with any subsequent advantage on creative performance.

## General discussion

The three experiments reported in this paper aimed to investigate the impact of a series of episodic and semantic inductions on divergent thinking. The dynamics of semantic operations underlying creativity have received a great deal of focus over many decades [35, 37, 39, 42], but the impact of episodic memory processes in creative thinking is now increasingly gaining traction [7, 95]. To date, only one published study has assessed both episodic and semantic memory operations within the same experimental paradigm in relation to creativity [34]. The present series of experiments was novel in that it concurrently examined the influence of both types of declarative memory operations using parallel induction methods compared to a non-declarative memory control induction as well as a no-induction baseline on creative performance, all within one paradigm. This is an important step forward as it is essential to evaluate the specificity of the involvement of episodic retrieval processes by comparing the effects of episodic induction with another non-episodic declarative memory induction as well as a non-declarative memory induction. Moreover, in order to evaluate whether inductions actually enhance creative thinking significantly more than not having an induction at all, the inclusion of a no-induction baseline is an important control to have in place, and it is one that has not been previously implemented within the creativity and memory domain. This enables us to identify whether the inductions augment, worsen, or have no effect on creative performance in comparison to a context in which there is no induction given prior to completing a creativity task.

The current study also considers the two approaches to measuring creativity or originality on divergent thinking tasks within the literature. One is an objective method and the other takes a subjective approach. While the objective method (calculated by the response frequency in a sample) is an increasingly employed method of scoring originality, it has been criticised when used on small samples [96]. Therefore, using a subjective method (judged by independent raters) to evaluate and identify uncommon ideas may be the more fitting approach to take in the present context [97]. On the other hand, the subjective method also suffers from critical shortcomings associated with interrater reliability and individual differences among raters in background knowledge and expertise. In light of these issues, the current study adopted both the objective and subjective scoring methods of creativity and is the first study to implement this approach in the memory and creativity domain.

### Memory induction and creative performance

The results across all three experiments revealed no significant impact on creative performance following either the episodic or semantic inductions compared to the control mathematical reasoning induction, and this was true for both younger and older adults. This indicates that no specific advantages or disadvantages were associated with any of the declarative memory inductions in that none enhanced or worsened creative performance.

It was predicted that the episodic and semantic inductions would lead to higher creative performance in comparison to the no-induction baseline for both younger and older adults. However, the pattern of findings across the three experiments did not support this prediction. The induction-based findings were limited and inconclusive as they differed across experiments and with respect to age. Compared to having no induction procedure prior to creative performance, there was evidence of semantic induction-related declines in fluency (older adults: Experiment 1) as well as control induction-related improvements in fluency (younger adults: Experiment 2). Importantly, there was no evidence that the episodic induction via the ESI procedure [7, 8] improved performance on any aspect of divergent thinking when compared to undergoing no prior induction at all.

It is to be noted that previous work adopting the ESI [7, 8] utilised a verification process to ensure that the induction, in comparison to a control induction, resulted in an increase in episodic details generated on subsequent tasks which rely on episodic memory compared to a task which does not. Whilst no such manipulation checks were in place for the induction procedures in the series of experiments reported here, we took a similar approach as the only published study examining the effect of episodic and semantic memory inductions on creativity in the same paradigm [34]. Just as in Beaty et al. [34] we aimed to prime an episodic or semantic retrieval orientation by engaging participants in relevant episodic or semantic tasks prior to the AUT. The tasks and scoring protocols chosen for the induction procedures were carefully selected on the basis that they have been extensively used in the literature to measure episodic and semantic memory operations. In addition, we have considered induction responsivity in our analyses to assess whether participants’ engagement with the tasks fit in with the literature as expected for their age.

A pertinent issue in the literature concerns the independence or interdependence of episodic and semantic memory within declarative memory. While much of the neuropsychological evidence support the notion of doubly dissociable components of declarative memory [e.g., 98, 99], the complete functional independence of episodic and semantic memory has been questioned since the inception of Tulving’s episodic theory [e.g., 23, 100–102]. Specifically, neuroscientific evidence indicates partially overlapping brain systems across a range of different episodic or semantic encoding and retrieval tasks [103, 104].

An important question related to this debate is the extent to which induction techniques can be ‘process pure’ in primarily targeting either episodic or semantic memory. For example, the free association task (Experiment 1) and the associative pathway task (Experiment 2) primarily engage semantic processing, but it cannot be ruled out that participants engaged episodic processes to some extent via cued retrieval of past experiences, which may have influenced the findings in the series of experiments reported here. Verbal episodic and semantic induction procedures will inevitably overlap to some extent regarding their operations by virtue of the fact that they are both part of the declarative memory system. While the details of this important discussion regarding process purity are beyond the scope of this paper, consideration of their implications is vital in the memory-creativity research context. This is because it questions whether the observed effects associated with a particular episodic specificity induction are only specific to the type of episodic induction implemented in a given study or are instead generalisable to wider dynamics of episodic memory retrieval processes as such. The current study has attempted to address this through the exploration of using diverse protocols of episodic and semantic inductions. No clear evidence was found for the effects being specific to the type of induction employed nor for the generalisability to wider dynamics of episodic processes. As such, it may be important to consider whether it is the ‘episodic’, ‘semantic’ or other elements of the induction that leads to enhancements in creative cognition as evidenced in previous work.

### Ageing and creative performance

The findings from the series of three experiments reported here reveal a relatively consistent effect of age such that older adults performed better than younger adults with regard to fluency (Experiments 3), average originality (Experiments 2 & 3), peak originality (Experiments 3), subjective creativity ratings (Experiments 2 & 3) and flexibility (Experiment 3) (see Table 4). This is contrary to Madore and colleagues [8] who found younger and older adults to perform comparably on the same divergent thinking task. The present study provides evidence for healthy older adults being more *fluent and flexible* as well as more *original* (scored using objective and subjective measures) in their responses on a divergent thinking task compared to younger adults. This was also the case in no-induction baseline comparisons in Experiments 2 and 3, suggesting that older adults performed better than younger adults even in the absence of inductions. Originality was the most consistent measure of the AUT across the three experiments in which older adults outperformed younger adults regardless of induction condition. This is especially noteworthy given that originality constitutes the defining attribute of creativity [105, 106].

**Table 4:** Visual presentation of the main effect of age across 3 experiments. In all cases, the main effect of age indicated that the older adults performed better than the younger adults on the measures in question.

	Fluency			Average Originality			Peak Originality			Subjective Creativity Rating			Flexibility		
ANOVA FINDINGS:	*Main effect of Induction*	*Main effect of Age*	*Age x Induction interaction*	*Main effect of Induction*	*Main effect of Age*	*Age x Induction interaction*	*Main effect of Induction*	*Main effect of Age*	*Age x Induction interaction*	*Main effect of Induction*	*Main effect of Age*	*Age x Induction interaction*	*Main effect of Induction*	*Main effect of Age*	*Age x Induction interaction*
*Experiment 1*	X	X	X	X	X	X	X	X	X	X	X	X	*Flexibility was not measured in Experiments 1 and 2*.		
*Experiment 2*	X	X	X	X	**✓**	X	X	X	X	X	**✓**	X			
*Experiment 3*	X	**✓**	X	X	**✓**	X	X	**✓**	X	X	**✓**	X	X	**✓**	X

The general consensus within research on creativity and ageing is that younger and older adults seem to perform comparably on divergent thinking tasks [12, 92], although there is some contradicting evidence suggesting that older adults perform poorer than young adults [93, 94]. The speed at which older adults process information is slower than younger adults [107]. According to Salthouse, this slowness is an essential mechanism that accounts for the majority of the age-related declines on several cognitive tasks, such as working memory and long-term memory. This suggests that older adults may in fact require more time to complete tasks and consequently, it is not known how this low processing speed or slow response time may have influenced older adult performance in timed conditions on divergent thinking tasks in past studies [11, 93]. Consistent with past studies, and the recent systematic review by Fusi et al. [e.g., 11, 12, 14], the experiments reported here confirm that when older adults are tested under conditions which do not restrict them (i.e., generous time constraints) they show preserved creative capacity, or even greater creative capacity than younger adults.

Brain-based models of creativity suggest that the default mode network is implicated in the generation of ideas from memory, whereas executive control networks are then engaged to inhibit salient ideas and to evaluate ideas [34]. In relation to ageing, data shows that older adults demonstrate reduced functional connectivity *within* the default and executive control networks [108, 109], but increased connectivity *between* the networks [110], leading to the *default-executive coupling hypothesis of ageing* [DECHA; 111]. The DECHA suggests that functional brain changes in older adults indicate greater coupling of lateral prefrontal cortex and the default network as cognitive control demands increase in older adults. These functional changes may reflect an adaptive shift in older adults as they begin to become more reliant on stored representations and crystalised cognitive abilities in order to support goal-directed tasks as a result of declined cognitive control capacities [112].

Recently, the implications of such functional changes with age have been explored in relation to creativity, where greater connectivity between default and executive networks may be beneficial for older adults [113]. Adnan et al. [113] examined activity and interactivity between default and executive control networks during the AUT. Consistent with previous studies implicating default and executive networks in divergent thinking in younger adults [114], they showed both younger and older adults demonstrate default-executive coupling during the AUT, yet despite comparable performance on the AUT, the strength of the network coupling differed between age groups. Older adults demonstrated greater default-executive connectivity compared to younger adults during the AUT. Critically, the results indicated that greater levels of default-executive coupling positively correlated with creativity ratings on the AUT for older adults only. Similar findings have been reported more recently by Patil et al. [115], who provided evidence consistent with the DECHA, showing age-related differences in functional connectivity associated with creativity. They suggest that these findings indicate age-specific changes in creative cognitive processes and demonstrate a beneficial shift in the neural architecture related to creative cognition in older adults. According to Adnan and colleagues [113] greater default network engagement may facilitate retrieval of prior knowledge to support divergent thinking in the context of declining cognitive control abilities in later life. It is therefore reasonable to postulate that the greater performance of older adults observed in the experiments reported here may reflect the age- related changes in functional connectivity [like that found by Patil and colleagues, 115], and the resulting enhanced reliance on knowledge and stored representations during creative idea generation.

Prior explanations regarding preserved creative capacity in older adults have also emphasised the wealth of experience held by older adults due to having a longer lifespan. Experiences with particular objects (stored in declarative memory) are reflected on with more depth and having a variety of experiences may potentially lead to improved performance on tasks that involve the use of materials and objects, such as the alternate uses task [11]. Evidence from research into the cognitive predictors of problem solving also points in the same direction. While younger adults primarily rely on fluid intelligence, older adults increasingly rely on knowledge or crystalised intelligence to support every day problem solving [116]. The accumulation of experiences and knowledge in later life may result in older adults having an advantage over younger adults in practical problem solving, when the to-be-solved problem is familiar to them [117]. Indeed, some studies indicate comparable or greater performance in older adults compared to younger adults in practical everyday problem solving [118]. These findings suggest that greater life experience is potentially the largest contributory factor in maintaining problem-solving performance in the context of ageing. However, other studies have shown poorer performance in older adults on practical problem-solving tasks [119]. Further work is therefore warranted to gain a fuller picture of practical problem solving in the context of ageing to be able to reach a meaningful conclusion in relation to the current findings in this regard.

An interesting issue in the context of practical problem solving and creativity is the dynamics of the facilitatory role of knowledge. The relationship between creativity and knowledge has been of interest for many decades [120]. The ‘foundation view’ argues for a linear positive correlation between the two and thereby posits that extensive knowledge is advantageous for creativity. The ‘tension view’, in contrast, characterises the relationship between creativity and knowledge as following an inverted-U function. Too little knowledge is insufficient for creativity whereas too much knowledge can lead to inflexibility. The middle ground is therefore ideal for creativity in the tension view as one has enough relevant knowledge but is not constrained in one’s ideation by having too much of it. Which of these views has the potential to best explain the findings of better performance of older adults compared to younger adults in creative problem solving is an open question. On one hand, it could be argued that the findings fit with the foundation view, as it could be postulated that older adults possess a greater wealth of practical knowledge than younger adults, which enabled them to come up with more creative responses. On the other hand, age-related declines in certain cognitive abilities may deplete the effects of extensive knowledge constraints on creativity. So, the alternative case can also be made, namely that inverted-U tension view may apply in the context of healthy ageing.

Following this line of thought, a related proposal can be made for age-related changes with respect to executive function abilities. One such change is diminished inhibitory control. Specifically, there is data showing poorer performance in older adults relative to younger controls on tasks measuring the ability to ignore distracting information, and the ability to inhibit the interference of dominant responses [121–123]. Reduced inhibitory control in older adults may prove beneficial in maintaining or enhancing creative capacity, by allowing conditions under which the processes necessary for creativity (e.g., overcoming knowledge constraints and conceptual expansion) can be efficaciously engaged in for creative idea generation. Previous work has shown that being able to apply novel additions to existing elements or perspectives (i.e., conceptually expand) and overcome the hindering effect of existing knowledge leads to more novel idea generation [124, 125]. Crucially, in relation to both conceptual expansion and the ability to overcome knowledge constraints, research suggests that deficiencies in cognitive inhibition contributes to greater performance in tasks demanding these cognitive processes [84]. Similarly, evidence also suggests that when resources for inhibition are depleted, the frequency and the originality of ideas is facilitated [126]. The reduced ability to ignore the incoming of irrelevant information in older adults may result in them being susceptible to the effects of distracting information. Carpenter, Chae and Yoon [126] investigated the impact of this on creative idea generation in younger and older adults. They found older adults generated more creative uses following the exposure of distracting information compared to younger adults. These findings further demonstrate that the inability to inhibit the processing of distracting information leads to enhanced creative performance. Together, the above lines of evidence suggests that relying on knowledge stored in declarative memory combined with a lack of inhibitory control in healthy older adults can enhance creative idea generation.

### Ageing, memory, and creative performance

Given the dissociation between episodic and semantic memory processes in healthy older adults, specifically the decline of episodic memory and preserved semantic memory [17], it was expected that inducing episodic and semantic memory processes may also have differentiating effects on the AUT as a function of ageing. The first study published to induce memory processes prior to completing the alternate uses task on older adults, found no significant interaction between age and induction on fluency, flexibility, or originality (as measured by subjective creativity ratings) [8]. The present study supports this finding in relation to the above measures of divergent thinking along with two further objective measures of originality (average originality and peak originality). This indicates that the episodic and semantic inductions have no differentiating effects on measures of the AUT as a function of age.

The logic proposed by Madore et al. [7, 8] in relation to the role of episodic memory on creativity was such that if a task relies on episodic memory processes, performance should be enhanced following an episodic induction. Therefore, a decline in episodic memory should result in a corresponding decline in creative performance. Indeed, some neuropsychological evidence supports this proposal, for instance, amnesic groups with a profound impairment of episodic memory have been found to exhibit impairments in verbal and visual creativity (2).

However, this explanation is not in line with the pattern of findings in the present study. A decline in episodic memory, associated with healthy ageing, is a robust finding in the ageing and memory literature [16, 17, 69] and yet, we did not see a corresponding impoverished creative output relative to young controls across the three experiments. Considering this, Madore and colleagues [8] suggested that the ESI may also tap onto processes such as visual imagery, which is preserved in older adults, which might explain why the ESI had a boosting effect for older adults on the AUT in their study. While they consider this as a possibility, the primary interpretation of their results is that the induction served to activate episodic retrieval processes in older adults too. With older adults performing better than younger adults on several measures of the AUT across several experiments, regardless of the type of declarative memory inductions and even in the absence of inductions, the case made for episodic memory processes does not contribute to explaining age-related effects in the current study. Alternative explanations must therefore be considered.

Intellectual ability may be one factor to consider. The relationship between intelligence and creativity has been extensively studied [see 127]. The threshold hypothesis of intelligence and creativity postulates a positive linear correlation between creativity and IQ, such that lower IQ is associated with lower levels of creativity [128]. In the present study however, older adult participants had lower IQ scores than the younger adults in two out of three experiments. Nonetheless, even after controlling for IQ in all three experiments, older adults outperformed the younger adults on several measures of the AUT.

One possible explanation for the present findings is drawn from research into the maintenance of verbal proficiencies in older adults, such as vocabulary, word comprehension and verbal working memory. It has been found that older adults are significantly better than young adults in verbal divergent thinking tasks, whereas the opposite is true for visual divergent thinking tasks [129]. It is therefore reasonable to postulate that verbal divergent thinking in older adults may be supported by the verbal proficiencies that remain intact across the lifespan [12].

The recruitment methods employed when adopting older adult samples in a series of studies may be a factor of consideration as well given that many of the older participants took part in two or more of the experiments reported here. It is important to note though that there was no linear increase in creative performance in older participants across the three experiments. Moreover, the samples were not identical across the three experiments and a whole new group of older participants took part in Experiment 3, eliminating the possibility of practice effects.

The amount of time available to participants to undertake the AUT may also have contributed to the findings of an age-based difference. Some previous work has indicated the need to consider the type of time constraints adopted in creativity research when adopting an older adult sample [11, 56]. The majority of studies employing the AUT provide 2 minutes for a single AUT trial. Older adults may need longer to perform cognitive tasks due to lower processing speed, which is often seen when testing fluency in older adults [107]. Following evidence which suggests that responses on creativity tasks produced later on in the response output are more creative than the responses produced early on [36, 57], the older sample may have had ample time in the current study to exhaust the initial responses and reach the more original and unique responses as no rigorous time constraints were in place.

One concern regarding the sample is the modest sample size and the absence of a priori power analysis to determine adequate sample size in order to observe an effect. Nevertheless, it is to be noted that the sample sizes obtained for each of the experiments were in line with those reported in previous induction-related ageing studies and induction and creativity ageing studies, where induction-related effects within age groups and overall age-related effects between groups have been observed [e.g., 3, 8, 18].

While we have found relatively consistent evidence for older adults demonstrating higher levels of creative performance than younger adults, the generalisability of these findings is limited. Participants engaged in a single task of divergent thinking across the three experiments, the alternate uses task. No other verbal or non-verbal tasks of creativity were included. The generalisability of the present study is therefore restricted to the context of the AUT as a measure of divergent thinking. A question for future exploration then, is whether these age effects are task specific or whether similar findings would surface using other measures of creative ideation.

## Conclusions

The findings of the experiments reported here reveal that (a) there is no evidence regarding a specific advantage associated with the induction of episodic or semantic declarative memory operations prior to creative performance, and (b) there is evidence to show that healthy ageing confers an advantage in creative performance in terms of greater fluency, originality, and flexibility during idea generation. The inclusion of a semantic induction and control non-declarative memory induction proved useful in revealing that effects of inductions are not specific to episodic memory in particular or declarative memory in general. The present study also showed the importance of including a no-induction baseline of creative performance in order to draw meaningful conclusions as to whether inductions improve, hinder, or are inconsequential to creative ideation when compared to having no prior induction. Moreover, it is apparent from the findings that research across a range of age groups is needed, given the evidence of clear age-based differences. Future work should explore whether such age-related effects follow a linear pattern when adopting intermediate age groups. Some studies have in fact adopted a similar approach [130, 131] and reported mixed findings with regards to the linear pattern of creative performance with respect to age. To evaluate the importance of factors such as life experience, wisdom, or practical knowledge in abetting divergent thinking performance, future investigations should include assessments of such factors so that a comprehensive picture on ageing and creativity can be developed.
